# Electronic patient-reported outcome systems and capabilities in cancer care: a systematic review

**DOI:** 10.3389/fdgth.2025.1560533

**Published:** 2025-08-18

**Authors:** Hosna Salmani, Somayeh Nasiri, Mahdi Alemrajabi, Maryam Ahmadi

**Affiliations:** ^1^ School of Health Management and Information Sciences, Iran University of Medical Sciences, Tehran, Iran; ^2^ Gastrointestinal and Liver Diseases Research Center, Iran University of Medical Sciences, Tehran, Iran

**Keywords:** electronic patient-reported outcome systems, ePRO, cancer, patient-centred solutions, capability

## Abstract

**Background:**

Cancer care increasingly emphasizes patient-centred approaches, leading to the adoption of electronic patient-reported outcome (ePRO) systems for essential patient data collection. Our systematic review investigates the landscape of electronic patient-reported outcome systems and their capability in cancer care, focusing on their potential to enhance patient-centred solutions.

**Methods:**

We conducted a systematic review, encompassing studies on electronic patient-reported outcomes in cancer. We searched in Scopus, Web of Science, and PubMed using comprehensive Medical Subject Heading (MeSH) terms up to April 2024. Papers were categorized based on nine key aspects, including author, publication year, country/state, objective, participants, cancer type, system name, system capabilities, and type of platform. Eligible studies were appraised using a mixed-methods appraisal tool (MMAT).

**Results:**

Analysis of 85 studies indicated a diverse range of electronic Patient-Reported Outcome systems and platforms in cancer care, Notably, PRO-CTCAE and CHES were frequently cited for their roles in data collection and analysis. Moreover, web-based platforms were predominant, followed by mobile-based and computer-based systems. In addition, Symptom assessment and management emerged as significant capabilities in the utilization of these systems for oncology care.

**Conclusion:**

Our systematic review of electronic patient-reported outcome (ePRO) systems in cancer care focused on the capabilities of these systems for capturing patient data and improving cancer treatment outcomes. This study emphasized the potential of electronic systems to enhance patient-centred oncology practices and optimize cancer care delivery.

## 1 Introduction

Cancer, with its complex treatment regimens and multifaceted impact on patients, demands a comprehensive approach to care that centres on the individual's experience and needs (1). In recent years, there has been an increasing recognition of the significance of integrating patient-reported outcomes (PROs) into oncology care to gain a deeper understanding of patients’ experiences, symptoms, and quality of life. Electronic Patient-Reported Outcome (ePRO) systems have emerged as potent tools in this endeavour, providing tools to collect, monitor, and analyze patient-reported data in real-time (1, 2).

Notwithstanding the progress in oncology care, a central challenge persists the need to address patients’ needs effectively, promptly, and comprehensively. Traditional methods of data collection often lack timeliness and depth, hindering clinicians’ ability to intervene proactively. Herein lies the crucial role of ePRO systems: they serve as a conduit for timely, accurate, and comprehensive data collection from patients, enabling clinicians to tailor interventions and support services accordingly (3).

Key electronic systems such as the Computer-Based Health Evaluation System (CHES) and electronic patient self-Reporting of Adverse-events: Patient Information and aDvice (eRAPID) facilitate proactive monitoring of patients’ symptoms and adverse events, enabling timely interventions and support services to optimize patient outcomes. Moreover, these systems empower patients to actively engage in their care by providing them with tools and resources to self-report their symptoms and communicate with their healthcare providers. This collaborative approach not only enhances patient satisfaction but also improves treatment adherence and clinical outcomes (4, 5). CHES has been widely embraced for their capacity to streamline data collection processes and provide clinicians with actionable insights into patients’ symptoms and treatment responses (6). CHES enables the seamless capture of patient-reported data through user-friendly interfaces, empowering clinicians to remotely monitor patients’ progress and adjust treatment plans accordingly (4). Similarly, eRAPID has played a pivotal role in enhancing patient engagement and symptom management in oncology care (5). This web-based system allows patients to report their symptoms in real-time and receive personalized advice and support from healthcare providers, leading to enhanced patient outcomes and satisfaction (7).

The integration of electronic health systems and platforms in oncology care holds significant promise for transforming patient-centred solutions. By leveraging the power of ePRO systems and ePROMs, clinicians can gain valuable insights into patients’ experiences, preferences, and treatment outcomes, allowing for more personalized and holistic care delivery (8, 9). In addition to ePRO systems, electronic Patient-Reported Outcome Measures (ePROMs) play a crucial role in capturing patients’ perspectives on their health and well-being (10–12). Platforms like Noona have been designed to facilitate the collection of ePROMs through web-based surveys and questionnaires, enabling clinicians to assess patients’ symptoms, functional status, and quality of life (13, 14). Noona's intuitive interface and customizable reporting features make it a valuable tool for tracking patients’ progress over time and identifying areas for intervention or support (15). Similarly, the EPIC Electronic Health Record (EHR) system provides robust capabilities for incorporating ePROMs into clinical workflows, enabling seamless integration of patient-reported data with other electronic health records (16).

Despite the increasing adoption of electronic health systems in oncology care (17), there exists a need for a comprehensive review of their capabilities and impact on patient-centred solutions. Existing studies have predominantly focused on individual systems or specific aspects of electronic health technology, constraining our understanding of their collective potential in oncology care delivery (18, 19). Furthermore, given the rapid technological advancements and evolving healthcare landscape, there is an urgent requirement for up-to-date research that synthesizes the current state of electronic health systems and platforms in oncology care while identifying areas for future development and enhancement.

Our systematic review aims to explore the landscape of electronic patient-reported outcome systems and platforms utilized in oncology care, with a specific focus on their capability to transform patient-centred solutions. By synthesizing existing literature and evaluating the combined capabilities of these systems, this study seeks to pinpoint knowledge gaps, showcase best practices, and provide recommendations for future research endeavours and implementation strategies. Through this undertaking, we aspire to contribute significantly to the ongoing discourse on the role of electronic health technology in oncology care with the ultimate goal of enhancing the quality of care delivery and outcomes for cancer patients.

The research questions for our systematic review could be formulated as follows:

RQ1. What are the electronic Patient-Reported Outcome (ePRO) systems and how do they contribute to enhancing patient-centred solutions in oncology care?
RQ2. What are the capabilities of ePRO systems and how do they capture patient experiences, symptoms, and treatment outcomes in cancer care?

These research questions will aid in systematically evaluating the effectiveness of ePRO systems in cancer care and identifying optimal approaches for their successful implementation and widespread adoption.

## 2 Methods

We conducted a systematic review concentrating on electronic patient-reported outcome (ePRO) systems and their potential for patient-centred solutions in the context of cancer care. The final report follows the Preferred Reporting Items for Systematic Reviews and Meta-Analyses (PRISMA) guidelines for reporting systematic reviews. Our study encompasses several key steps including search strategy, inclusion and exclusion criteria, study selection, quality appraisal, and data extraction and synthesis to ensure a comprehensive and rigorous analysis of the available literature (20).

### 2.1 Search strategy

We conducted our search by exploring academic articles across electronic databases such as Scopus, Web of Science, and PubMed. We searched Scopus, Web of Science, and PubMed using comprehensive Medical Subject Heading (MeSH) terms up to April 2024. The search strategy involved the use of specific keywords and medical subject heading (MeSH) terms focusing on two overarching themes: “electronic Patient Reported Outcomes” and “Cancer” to identify relevant studies. Additionally, a backward snowball search technique was employed to enhance the scope of our search. Following this process, all identified studies were imported into EndNote software to manage duplicates effectively. (See Supplementary Appendix A).

### 2.2 Inclusion and exclusion criteria

In this study, the inclusion and exclusion criteria are as follows:

Inclusion Criteria
  1. Language: English language papers will be included.
  2. Publication Type: Articles published in peer-reviewed journals or presented at reputable conferences that have full text will be included.
  3. Study Designs: Quantitative, qualitative, and mixed-methods studies will be considered.
  4. Topic Relevance: Studies focusing on electronic patient-reported outcomes (ePROs) will be included.
Exclusion Criteria
  1. Technical Infrastructure Studies: Studies primarily focusing on technical infrastructure or software development unrelated to patient-reported outcomes will be excluded.
  2. Paper-Based Assessments: Studies emphasizing paper-based patient-reported outcome measures will be excluded.
  3. Irrelevant Topics: Studies not related to cancer or lacking clear indicators or outcomes pertinent to cancer care will be excluded.

### 2.3 Study selection

In our study selection process, two reviewers independently assessed the titles and abstracts of identified papers based on predefined criteria, resolving any disagreements with a third reviewer. After excluding irrelevant studies, one reviewer conducted data extraction, cross-verified by other team members for accuracy. This process occurred in two steps: initial screening of titles and abstracts, followed by full-text review. Both stages were independently carried out by two reviewers, with final inclusion contingent upon consensus to ensure the inclusion of only the most relevant research.

### 2.4 Quality assessment

The quality of the selected articles was evaluated using the Mixed Methods Appraisal Tool (MMAT), a comprehensive instrument designed to assess the methodological rigour of various types of research studies. The MMAT encompasses specific criteria tailored for qualitative, quantitative clinical trials, non-clinical trials, descriptive studies, and mixed methods research. Each article was categorized based on its study type, and the corresponding criteria were applied accordingly. During the assessment, reviewers responded to screening questions and rated the criteria within the chosen category. The “Can't tell” response category was utilized when the paper lacked sufficient information for evaluation. Articles were categorized based on the percentage of positive responses obtained during the quality assessment (21).

### 2.5 Data extraction

An initial data extraction form was developed to capture key information from the selected studies. Data elements were organized into general (author, year, country/state, objective, and participants) and specific items (cancer type, system name, system capabilities, type of platform) to facilitate a comprehensive analysis.

### 2.6 Data synthesis

Thematic analysis was employed to identify common themes across the literature, with a focus on systems and capabilities for patient-centred solutions associated with electronic patient-reported outcome systems in cancer care. To ensure validity, extracted themes were cross-checked and discussed by the study authors. The synthesized data, including frequencies and percentages of Electronic Systems and Platforms and capabilities, were presented in tables and figures, contributing to a better understanding of ePRO systems in cancer care. The selected papers were summarized in the final step of our methodology, and important factors were identified. (See Supplementary Appendix B).

## 3 Results

### 3.1 General findings

**Figure 1:**
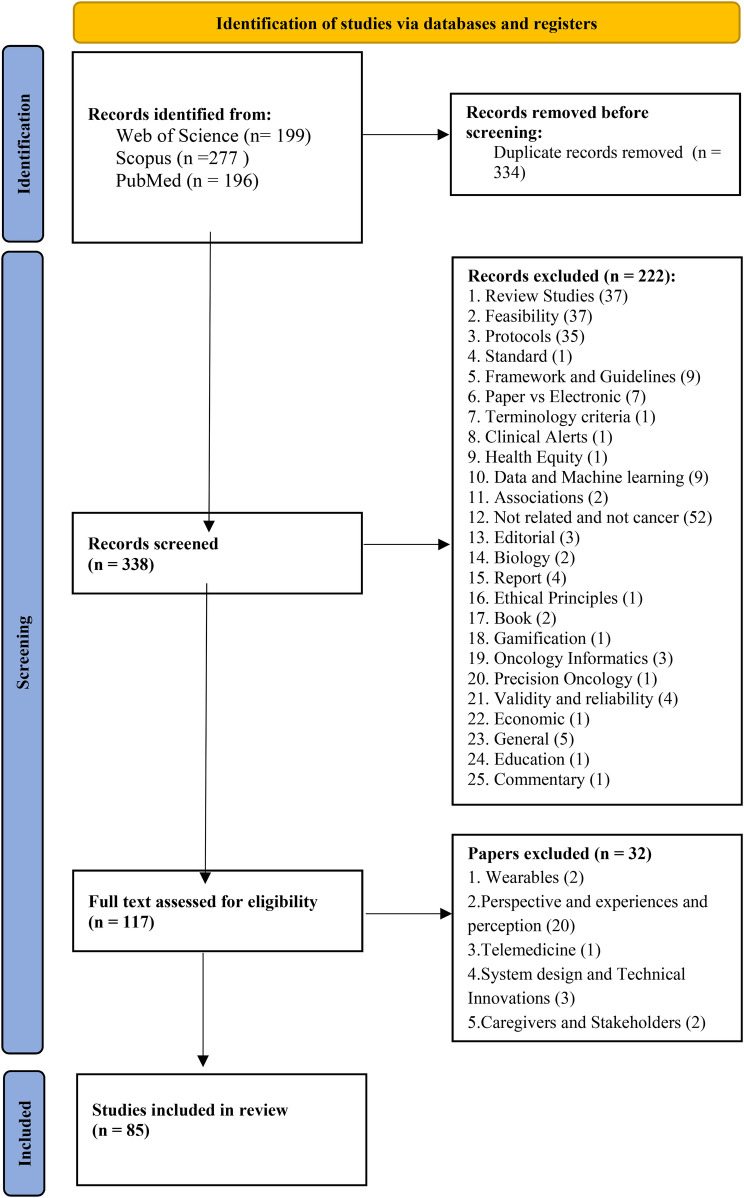
PRISMA flow diagram for selected studies.

In our systematic review, we identified 672 papers, out of which 85 academic papers were included in our systematic review, providing a comprehensive exploration of electronic health platforms for cancer care. we present the key findings regarding the characteristics of the included studies, electronic systems and platforms, and their system capabilities as revealed in our systematic review. (See Figure 1).

### 3.2 Characteristics of included studies

**Figure 2:**
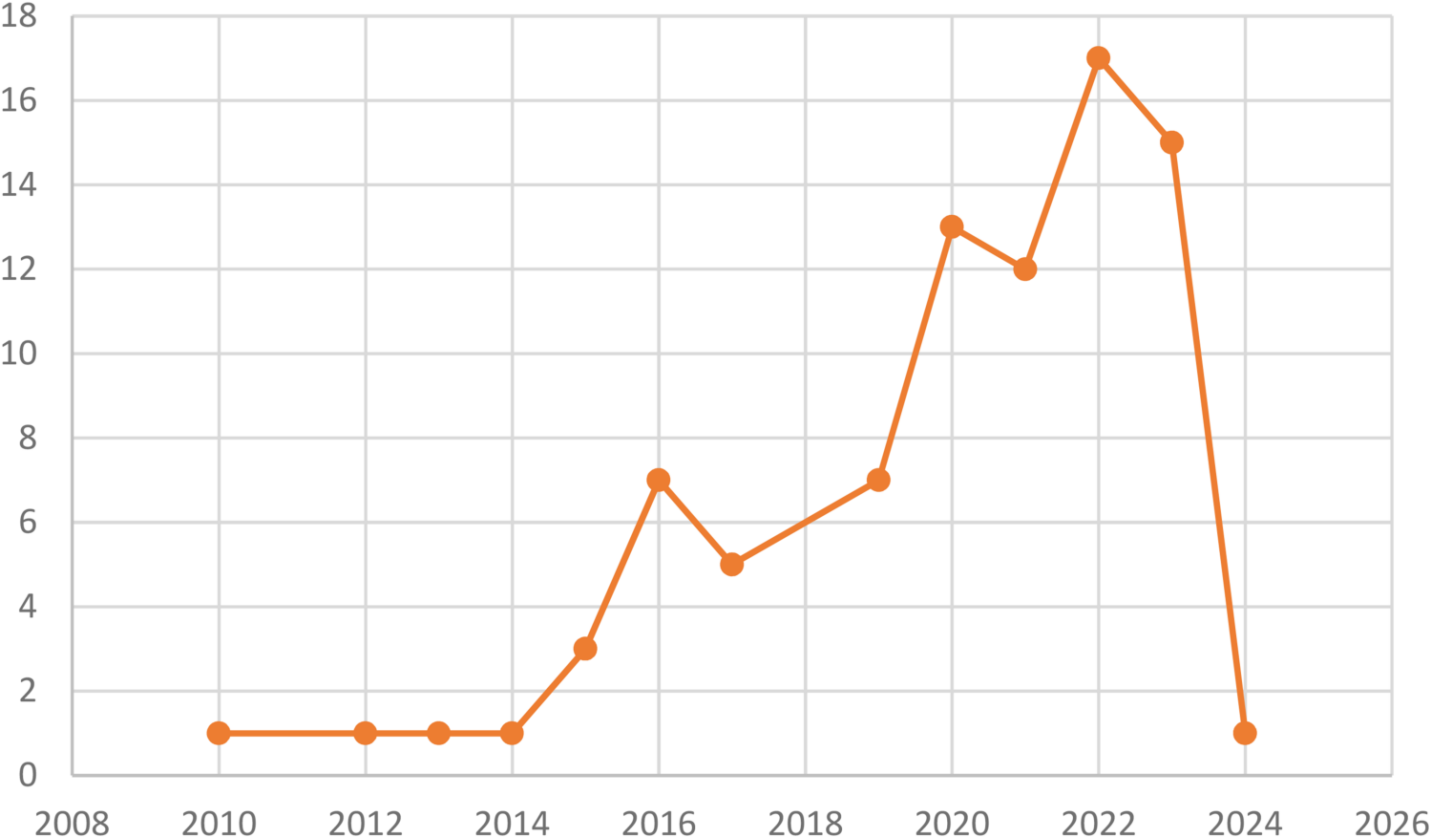
Distribution of documents by year.

The distribution by year indicates that the majority of publications were from the years 2022 and 2023, with 17 and 15 contributions, respectively. Additionally, there were 12 publications from 2021, 13 from 2020, 7 from 2019, 7 from 2016, 5 from 2017, 3 from 2015, and 1 publication each from 2024, 2014, 2013, 2012, and 2010. (See Figure 2).

**Figure 3:**
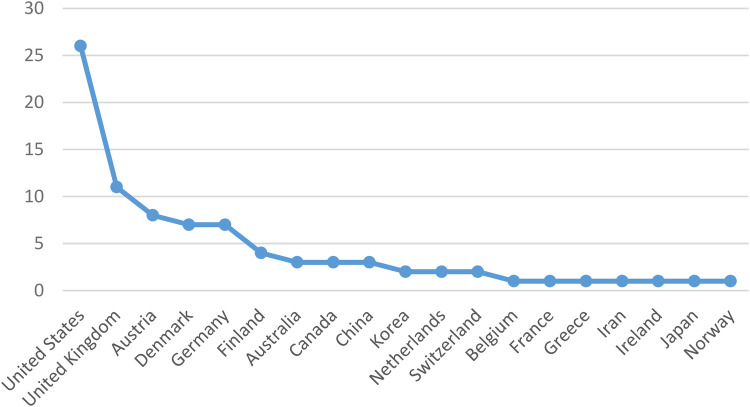
Source of publications by country.

According to Figure 3, the United States emerged as the leading source of publications, followed by the United Kingdom in second place and Austria in third. Additionally, Belgium, France, Greece, Iran, Ireland, Japan, and Norway each made a single contribution.

**Figure 4:**
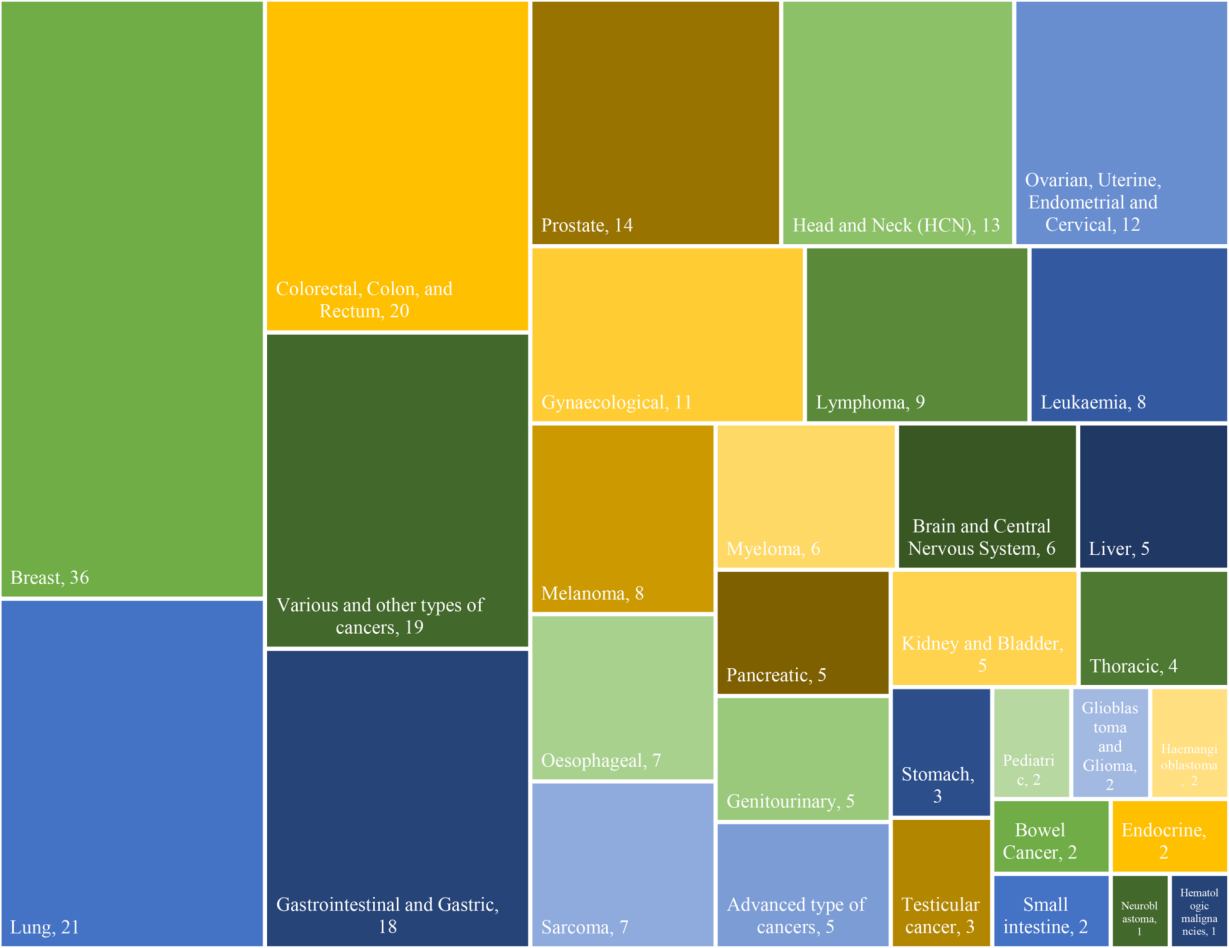
Frequency of Cancer Types in ePRO.

In terms of cancer types, a total of 32 different types of cancers, comprising 264 cases, were identified in the documents. The frequency distribution of cancer types is crucial as it highlights the prevalence of various malignancies across the studies. “Breast cancer” emerged as the most frequently studied, appearing in 36 articles, signifying its prominence in the literature (5, 7, 18, 22, 24, 25, 28, 33–35, 38, 39, 41, 43, 45, 47, 49, 52, 54–57, 60, 63–66, 72, 73, 76, 81–83, 87, 93, 96). “Lung” cancers (11, 14, 22, 24, 28, 34, 35, 38, 41, 44, 46–48, 54, 55, 60, 68, 72, 90, 93, 94) closely follow, sharing the second-highest frequency, represented by 21 studies. “Colorectal, (5, 7, 24, 28, 34, 35, 38, 41, 54, 60, 81, 83, 84, 86) colon, (22, 55, 66) and rectum” (18, 22, 55) cancers, also with a frequency of 20 cases, occupy the third-highest position. “Gastrointestinal (11, 15, 25, 29, 39, 45, 65, 72, 81, 90, 93, 96) and Gastric” (34, 35, 38, 41, 55, 65) cancers are shown as the fourth-highest, each with 18 occurrences. The Various and other cancer types collectively account for 16 instances, (4, 13, 16, 19, 22, 25, 35, 40, 41, 55, 58, 74, 88, 91) showcasing the diverse range of cancers and research focus. Furthermore, advanced types of cancers are represented by 5 studies. (15, 34, 36, 38, 53) Moreover, “neuroblastoma” (88) and “hematologic malignancies” (25) emerge as the least frequent cancer types, each documented in a single case. This analysis not only provides an overview of the distribution of cancer types but also highlights variations in research emphasis, emphasizing the need for a comprehensive understanding of different malignancies in the oncological landscape. The type and frequency of cancer within the study are indicated in Figure 4.

### 3.3 Electronic systems and platforms

**Figure 5:**
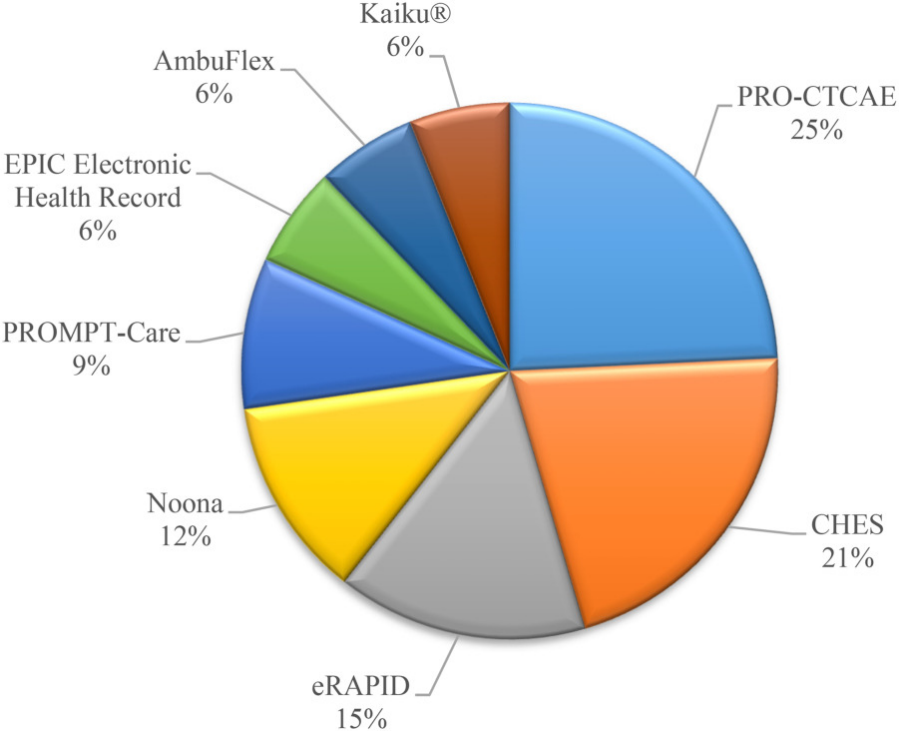
The distribution of selected studies according to the most famous system.

Based on the information gathered from various electronic Patient Reported Outcome Systems and platforms used in healthcare settings, it is evident that the frequency of utilization varies among different systems. Among the systems mentioned, PRO-CTCAE, a Patient Reported Outcome system, stands out with a frequency of 8. This system focuses on Patient-Reported Outcomes and is crucial for assessing treatment-related toxicity and therapeutic effectiveness (18, 19, 39, 51, 54, 57, 62, 74). Following closely is CHES (Computer-based Health Evaluation System) with a frequency of 7. CHES provides a comprehensive approach for electronic data capture and patient-reported outcome interpretation, making it a valuable tool in healthcare settings (4, 22, 55, 73, 90, 94, 95). In contrast, systems like eRAPID (*N* = 5) (5, 7, 29, 81, 83), Noona (*N* = 4) (13–15, 49), PROMPT-Care (*N* = 3) (44, 48, 69), EPIC Electronic Health Record (EHR) (*N* = 2) (16, 60), AmbuFlex (*N* = 2) (37, 84), Kaiku® (*N* = 2) (74, 85), and others have lower frequencies ranging from 5 to 1. Figure 5 shows the most popular repeated systems and platforms in studies.

In summary, while PRO-CTCAE and CHES emerge as more frequently mentioned systems in the sources, other platforms like eRAPID, Noona, and PROMPT-Care have lower frequencies but still play essential roles in electronic patient self-reporting and care management. Each system serves specific purposes within the healthcare landscape, contributing to improved patient care and outcomes. We provide the distribution of frequency of Electronic Systems and Platforms and their definition and usage of them in Supplementary Appendix C.

### 3.4 Types of ePRO platforms

Our study identified four types of platforms: Web-based, Mobile-based, Computer-based, and Web-based applications. Among the types of ePRO platforms in cancer, the most frequently mentioned category is Web-based platforms, with a total of 32 references (5, 7, 16, 25, 26, 29, 36–38, 42–44, 48, 51, 54, 56, 57, 59, 60, 62, 68, 69, 77–81, 83, 84, 89, 92, 93). Following closely is the Mobile-based category, cited 20 times. (15, 18, 19, 23, 27, 28, 30, 31, 33, 34, 41, 45, 50, 61, 66, 67, 71, 87, 88, 96) In contrast, Computer-based platforms are mentioned 16 times (4, 11, 22, 35, 39, 55, 64, 72, 73, 75, 76, 86, 90, 91, 94, 95). Interestingly, the least represented category among these ePRO platforms in cancer is the Web-based application type by 9 references (13, 14, 40, 49, 58, 65, 74, 82, 85). Moreover, 8 studies haven’t mentioned the system types and they only use ePRO systems (24, 32, 46, 47, 52, 53, 63, 70).

### 3.5 System capabilities

The evaluation of various system capabilities in oncology-focused electronic patient-reported outcome (ePRO) systems revealed a diverse range of functionalities and benefits across different domains. The study analyzed the frequency of different capabilities and features of electronic systems and platforms used in cancer care. To facilitate analysis and interpretation, the system capabilities were classified into distinct thematic categories based on their primary functions and roles in supporting patient care and research. These categories are defined as follows:

• **Symptom Assessment and Management:** Systems designed to detect, monitor, and manage patient symptoms, including the use of personalized symptom questionnaires and automated guidance for mild symptoms.
• **Remote Monitoring and Supportive Care:** Capabilities that enable continuous observation of patients’ health status remotely, allowing timely interventions and sustained supportive care.
• **Communication and Patient Engagement:** Features that promote interactive communication between patients and healthcare providers, enhance patient compliance, and support active participation in care.
• **Research and Clinical Trial Capabilities:** Functionalities supporting research data collection, patient eligibility screening, and integration into clinical trial workflows.
• **Data Visualization and Reporting:** Tools that transform patient-generated data into visual formats such as graphs or dashboards to support clinical interpretation and decision-making.
• **Usability and User Experience:** Assessments and features focused on ease of use, user satisfaction, and intuitive interface design for both patients and healthcare professionals.
• **Application Integration and Interoperability:** Capabilities enabling the exchange of data with Electronic Health Records (EHRs) and compatibility with third-party platforms, including mobile and social media applications.
• **Quality of Life and Psychological Factors:** Assessment modules that measure patient-reported quality of life, psychological well-being, distress levels, and related psychosocial indicators.
• **Data Security and Encryption:** Mechanisms for ensuring the secure collection, storage, and transfer of sensitive patient data in compliance with data protection regulations.
• **Patient-Reported Outcomes (PRO) Utilization:** Functions that facilitate the collection, analysis, and clinical use of patient-reported outcomes to inform care decisions.
• **Patient Recruitment and Enrollment:** Tools that assist in identifying, recruiting, and enrolling patients into clinical programs or research studies.

The most frequently studied capability was Symptom Assessment and Management, with 12 references (14, 16, 18, 19, 22, 25, 26, 29, 32, 33, 35, 36), followed by Remote Monitoring and Supportive Care, with a frequency of 9 references (14, 24–26, 28, 39, 40, 57, 96). This functionality enables early detection and management of symptoms, personalized symptom questionnaires, and automated standardized advice for mild symptoms.

**Figure 6:**
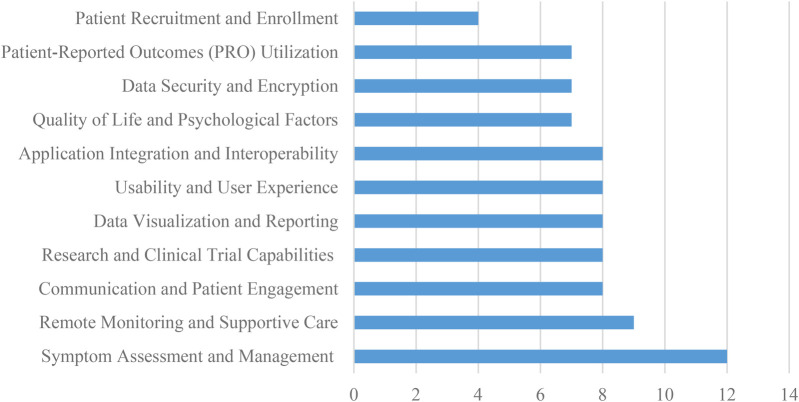
The frequency distribution of system capabilities in ePRO in Cancer.

Communication and Patient Engagement (18, 24, 26, 29, 38–40, 96), Research and Clinical Trial Capabilities (18, 24, 29, 38–40, 84, 96), Data Visualization and Reporting (18, 19, 25, 29, 36, 40, 84, 96), Usability and User Experience (18, 24, 26, 29, 38, 45, 84, 96), Application Integration and Interoperability (14, 18, 22, 24, 25, 28, 38, 96), all had 8 studies. Quality of Life and Psychological Factors (16, 19, 25, 26, 32, 33, 45), Data Security and Encryption (16, 18, 29, 33, 39, 45, 96), and Patient-Reported Outcomes (PRO) Utilization (18, 24, 29, 39, 40, 84, 96) all had 7 studies. These are also essential capabilities that can improve the quality of care and patient outcomes. The least studied capability was Patient Recruitment and Enrollment, with only 4 references (11, 16, 22, 96). For example, Communication and Patient Engagement can facilitate two-way communication between patients and caregivers, compliance and active participation tracking, and real-time communication between patients and clinicians (18), while Quality of Life and Psychological Factors can help evaluate quality of life, functionality, needs, distress, and fear of progression (11). Application Integration and Interoperability can facilitate seamless data exchange with Electronic Health Record (EHR) systems (14) and integration with popular social apps like WeChat for patient convenience (38). Data Security and Encryption ensures secure electronic data collection, while Patient-Reported Outcomes (PRO) Utilization can help collect patient-reported outcomes data and visualize patient responses to clinicians (40). Figure 6 shows the distribution of system capabilities in ePRO in Cancer.

In summary, the study found that Symptom Assessment and Management and Communication and Patient Engagement were the most frequently studied capabilities in electronic systems and platforms used in cancer care. Patient Recruitment and Enrollment was the least studied capability. Table 1 shows the overview of system capabilities in ePRO in cancer.

**Table 1:** Comprehensive overview of ePRO system capabilities in cancer.

Category	Frequency	Percentage	Capabilities
Symptom assessment and management (14, 16, 18, 19, 22, 25, 26, 29, 32, 33, 35, 36)	12	13.95%	- Symptom assessment, medication management, communication, and alerts. - Personalized symptom questionnaires categorizing health states. - Weekly symptom monitoring, and image recognition for Immune-Related Adverse Events (irAE) evaluation. - Automated standardized advice for mild irAEs, immediate alert for severe irAEs. - Patient self-reporting of symptoms during cancer therapy. - Real-time transfer and display of patient responses. - Home-based ePRO assessments for patient convenience. - Clinician feedback during clinical encounters.
Remote Monitoring and Supportive Care (14, 24–26, 28, 39, 40, 57, 96)	9	10.47%	- Web-based platform for remote symptom monitoring for patients undergoing radiation therapy. - Remote completion and in-house assessments with different completion rates for various cancer types. - Delivery of educational content for subjective care improvement. - Web-mediated application (Noona) for remote monitoring of patients with cancer. - Immediate automated advice for AE management and email notifications for severe AEs. - Real-time access to results and comparison of individual patient results with peer groups. - Enhancing patient empowerment with tailored self-management advice.
Communication and Patient Engagement (18, 24, 26, 29, 38–40, 96)	8	9.30%	- Two-way communication between patients and caregivers - Compliance and active participation tracking. - Collection of real-world data on patient symptoms during treatment. - Longitudinal assessment of treatment-related side effects. - Real-time communication between patients and clinicians. - Delivery of self-care instructions targeted to reports of moderate-severe symptoms. - Integration of electronic systems with clinical algorithms for patient advice and clinician alerts. - Integration of WeChat for patient reporting and convenience.
Research and Clinical Trial Capabilities (18, 24, 29, 38–40, 84, 96)	8	9.30%	- Assessing electronic systems’ capability to capture patient-reported outcome measures in clinical trials. - Integration of e-PRO data for personalized patient management in research. - Real-time research-quality data collection for clinical trials. - Immediate access to Adverse Event (AE) reports for clinical teams. - Generation of alerts for severe AEs, prioritizing critical cases for immediate attention. - Integration of PRO data into patient care. - Real-time, an online collection of PROs in mixed mode (paper or web-based). - PRO-based automated decision algorithms with defined thresholds. - Validation of HRQoL questionnaires and generation of electronic PROfiles.
Data Visualization and Reporting (18, 19, 25, 29, 36, 40, 84, 96)	8	9.30%	- Real-time graphic displays of e-PRO results for clinicians. - Data visualization for symptom change over time. - Access to information for self-management with ease of reporting symptoms via smartphones. - Generation of electronic profiles with normalized t scores for distress and despair. - Efficient tracking of symptom item changes over time. - Real-time electronic collection of patient-reported QOL data with color-coded reports. - Integration with Qualtrics for data capture, analysis, and de-identification.
Usability and User Experience (18, 24, 26, 29, 38, 45, 84, 96)	8	9.30%	- Ease of reporting symptoms via smartphones. - User-friendly design for effective information management. - Customizability of ePRO questionnaires for personalized patient experience. - Comprehensive training and evaluation for research assistants for smooth operation of the system. - Web-based platform for depression and anxiety assessment. - Compatibility with EHR integration. - Adaptable to institutional preferences. - Inclusion of oncology-specific modules. - Dot phrases for efficient and standardized documentation.
Application Integration and Interoperability (18, 22, 24, 25, 28, 38, 96)	8	9.30%	- Integration of ChemoPRO® with ChemoCare® platform for comprehensive care. - Seamless data exchange with EPIC EHR system. - Integration with popular social apps like WeChat for patient convenience. - Real-time survey monitoring with automated reminders and alerts. - Integration with Electronic Health Record (EHR) for streamlined operations. - Compatibility with institutional preferences for smooth implementation. - Remote symptom assessment with automated alerts.
Quality of Life and Psychological Factors (16, 19, 25, 26, 32, 33, 45)	7	8.14%	- Evaluate quality of life, functionality, needs, distress, and fear of progression. - Monitoring health status changes, and identifying risk factors. - Assessment of psychosocial factors, post-traumatic growth, resilience, and financial stress affecting HRQoL. - Efficiently collect HRQoL data suitable for breast cancer patients. - Daily ePRO assessments with red and yellow alerts. - Symptom fluctuation tracking over a week with association of alerts with future acute care events. - Monitoring physical activity and identifying patients in need of supportive care.
Data Security and Encryption (16, 18, 29, 33, 39, 45, 96)	7	8.14%	- Data encryption for secure electronic data collection. - Mobile-based platforms ensuring secure data transmission. - Secure real-time integration with Electronic Patient Records (EPR). - Patient-centered system with real-time symptom tracking. - Immediate care for high-risk patients with data stratification by risk level. - Real-time, online collection of patient-reported outcomes with self-assessment of side effects and quality of life. - Secure data transmission through NFC technology.
Patient-Reported Outcomes (PRO) Utilization (18, 24, 29, 39, 40, 84, 96)	7	8.14%	- Utilization of ePROs for patient triage and personalized care allocation. - Reduction in unnecessary consultations through patient empowerment. - Designed to collect patient-reported outcomes data and visualize patient responses to clinicians. - Collection of ePROs using validated HRQoL questionnaires. - Patient empowerment through direct contact with healthcare professionals. - Collection of patient-reported symptom data with the feasibility of e/Tablet use. - Significant impact on hospital utilization and cost of care with ePRO implementation.
Patient Recruitment and Enrollment (11, 16, 22, 96)	4	4.65%	- Efficient participant recruitment through the EPIC EHR system. - Seamless data collection facilitating enrollment. - Enhanced assessment of psychological distress for personalized care. - Early symptom detection and management. - Improved patient-provider communication through integrated systems. - Real-time data exchange between patient terminals, clinician terminals, clinical servers, and researcher terminals. - Alert generation for timely patient engagement.

## 4 Discussion

In our study, we conducted a systematic review aimed at synthesizing literature concerning electronic systems and platforms in oncology care. Analysis of publication distribution by year revealed that the majority of articles were published in 2022 and 2023, with the United States being the primary source of publications. Breast cancer emerged as the most frequently studied cancer type. Our focus was to comprehend the diverse capabilities of these systems in improving patient outcomes and streamlining healthcare processes. Specifically, we investigated the contributions of electronic Patient-Reported Outcome (ePRO) Systems and platforms in data capture, storage, and real-time analysis, tailored to researchers, clinicians, and patients. Additionally, we explored how these systems facilitate patient engagement in their care through symptom reporting and monitoring.

### 4.1 Electronic systems and platforms

The development of electronic systems in oncology care reflects a clear temporal progression aligned with advances in digital health. Initial ePRO systems were largely desktop-based, focusing on basic symptom tracking through static interfaces, often utilizing dedicated computer programs or early personal digital assistants (PDAs) for data capture (97). Over time, a significant shift occurred toward web-based platforms that allowed for greater accessibility, remote data entry, and enhanced clinician-patient interaction beyond the clinical setting (98). The widespread proliferation of mobile devices led to the next major transition, mobile-enabled ePRO apps, which offered increased convenience, portability, and real-time data capture from patients in their daily environments, facilitating continuous monitoring and immediate feedback (99). More recent systems emphasize advanced interoperability with Electronic Health Records (EHRs), streamlining data flow and integration into clinical workflows. These modern platforms also incorporate sophisticated capabilities such as automated alerts for worsening symptoms, adaptive symptom questionnaires that adjust based on patient input, and seamless telehealth integration to support comprehensive remote care (100).

Furtheremore, Several electronic patient-reported outcomes (ePRO) systems play a crucial role in transforming oncology care by providing advanced functionalities tailored to patient needs. Among these systems, CHES (Computer-based Health Evaluation System) stands out for its comprehensive approach to PRO assessment and data interpretation. Developed by Evaluation Software Development (ESD), CHES provides a convenient software solution for electronic data capture, storage, and analysis, facilitating the interpretation of patient-reported outcomes with graphical real-time feedback. This system provides a multicomponent approach adaptable to the specific requirements of researchers, clinicians, patients, and organizational settings (4, 22, 55, 73, 90, 94, 95).

Another notable system is Noona, a web app and patient outcomes management solution designed to engage patients in their care through real-time symptom reporting and monitoring. Noona facilitates streamlined clinical workflows, evidence-based care promotion, and access to rich data insights for better management throughout the care continuum. With features like access to clinical care teams, structured data capture with actionable content, and rapid deployment capabilities for immediate patient impact, Noona enhances communication between patients and healthcare providers while collecting essential data on patients’ symptoms at different phases of cancer care (13–15, 49).

Additionally, the EPIC Electronic Health Record (EHR) system plays a vital role in enhancing patient engagement and facilitating remote care. Epic's cloud-based EHR solution caters to various specialities and provides a range of core EHR features with the flexibility to add speciality-specific modules. With a strong focus on patient engagement, Epic provides extensive patient portal features, and telehealth options, and supports video visits and post-surgical follow-ups. By evaluating these systems’ capabilities in patient recruitment, symptom assessment, quality of life evaluation, communication, data security, research capabilities, data visualization, usability, and remote monitoring, we gained insights into their collective impact on improving oncology care delivery. This system is widely used across different healthcare practices, from community hospitals to multi-speciality hospital groups (16, 60).

Furthermore, eRAPID (Electronic patient self-Reporting of Adverse-events: Patient Information and aDvice) is another significant ePRO system that focuses on patients’ self-reporting of adverse events. This web-based system allows patients to complete symptom reports from home or mobile devices and receive severity-based advice. eRAPID enhances patient engagement by enabling individuals to report their symptoms in real-time, providing relevant information and advice based on their reported experiences for proactive symptom management (5, 7, 29, 81, 83).

In the realm of personalized cancer treatment and care, PROMPT-Care (Patient Reported Outcome Measures for Personalized Treatment and Care) plays a crucial role in collecting patient-reported outcome measures. This system is instrumental in tailoring cancer treatment plans based on individual patient needs and experiences (44, 48, 69). Additionally, PRO-CTCAE (Patient Reported Outcome of Common Terminology Criteria for Adverse Events) focuses on capturing patient-reported adverse event data to enhance symptom management and personalized care in oncology settings (18, 19, 39, 51, 54, 57, 62, 74).

Each of these systems demonstrates the potential to transform oncology care, providing innovative approaches to enhance patient-centred solutions. In summary, ePRO systems have proven beneficial in enhancing patient-centred care, improving symptom assessment and management, and optimizing quality of life and psychological factors in oncology care. Briefly, these advanced electronic systems play a crucial role in transforming oncology care by improving patient outcomes, enhancing communication between patients and healthcare providers, ensuring data security, facilitating research capabilities, and providing user-friendly interfaces for efficient information management.

In terms of system and platform type, the distribution of mentions across different types of ePRO platforms in cancer sheds light on the prevalent utilization patterns within healthcare settings. The Web-based platforms, as evidenced are the frequent type of platforms that have been used, indicate the significance of online interfaces in facilitating patient-reported outcomes in oncology care. This emphasis on Web-based solutions aligns with the increasing trend towards digital health technologies and remote patient monitoring (5, 7, 16, 25, 26, 29, 36–38, 42–44, 48, 51, 54, 56, 57, 59, 60, 62, 68, 69, 77–81, 83, 84, 89, 92, 93).

In contrast, the Mobile-based category is the second and reflects the growing adoption of mobile applications to enhance patient engagement and data collection in cancer care. The portability and accessibility of mobile platforms provide unique advantages in capturing real-time patient-reported data and promoting continuous monitoring outside traditional healthcare settings (15, 18, 19, 23, 27, 28, 30, 31, 33, 34, 41, 45, 50, 61, 66, 67, 71, 87, 88, 96). Interestingly, Computer-based platforms, despite being foundational in healthcare technology, indicate a relatively lower prevalence compared to Web-based and Mobile-based solutions (4, 11, 22, 35, 39, 55, 64, 72, 73, 75, 76, 86, 90, 91, 94, 95).

The least represented category, Web-based application platforms, highlights a niche area within ePRO systems in cancer care. While these platforms may provide specific functionalities or targeted solutions, their lower frequency of mention suggests a narrower focus or limited adoption compared to other types of ePRO platforms (13, 40, 49, 58, 65, 74, 82, 85). Generally, the distribution of mentions among different ePRO platform categories indicates the dynamic landscape of electronic systems in oncology care, preferences. This observation may suggest a shift towards more agile and user-friendly electronic systems that align with modern healthcare delivery models.

### 4.2 System capabilities

The section on electronic systems and platforms in oncology care reveals several key themes. These include symptom assessment and management, emphasizing real-time feedback for improved care delivery; communication and patient engagement, facilitating active involvement in the care process; research and clinical trial capabilities, supporting efficient data collection and analysis; data visualization and reporting, aiding comprehensive decision-making; usability and user experience, ensuring ease of use for all stakeholders; remote monitoring and supportive care, enhancing accessibility and continuity of care; quality of life and psychological factors assessment, enabling holistic care approaches; application integration and interoperability, promoting seamless functionality across systems; data security and encryption, ensuring confidentiality and privacy; patient-reported outcomes utilization, aiding personalized care and treatment decisions; and patient recruitment and enrollment streamlining, improving efficiency in research participation.

The integration of electronic patient-reported outcome (ePRO) systems in oncology care has significantly enhanced patient-centred solutions through various capabilities. These systems have transformed patient recruitment and enrollment processes by efficiently utilizing Electronic Health Records (EHR) systems, enabling seamless data collection and early symptom detection (25). The ability to recruit and enrol patients in ePRO systems is crucial for their successful implementation (16, 22). Studies such as those conducted by Riedl et al. (22), Sprave et al. (23), and Patt et al. (24) highlighted the significance of understanding patient demographics and preferences. Moreover, the integration of ePRO systems with popular social apps like WeChat has facilitated real-time survey monitoring and automated reminders for enhanced patient convenience and engagement (38). Studies from Andrew Harper et al. (25), Oldenburger et al. (26), and others demonstrated the integration of ePROs into routine care. These studies indicated the potential of ePRO systems to integrate into existing healthcare infrastructure, enhancing communication and data exchange.

Symptom assessment and management have been greatly improved through personalized symptom questionnaires, weekly symptom monitoring, and immediate alerts for severe adverse events (25, 53, 54, 86, 87, 96). These advancements allow for timely intervention and personalized care plans based on patients’ health states and symptom severity levels (29). Studies such as those by Andrew Harper et al. (25), Eva Oldenburger et al. (26), and others explored the impact of ePROs on symptom severity. Harper et al. (25) evaluated the electronic collection of patient-reported outcomes data across ambulatory cancer centres, while Oldenburger et al. (26) described the symptom severity among adolescents and young adults. These findings reinforce the role of ePROs in enhancing symptom assessment, reducing hospitalizations, and improving patient outcomes.

Additionally, the assessment of quality of life and psychological factors has been optimized through the collection of patient-reported outcomes, enabling the evaluation of distress, fear of progression, and post-traumatic growth affecting Health-Related Quality of Life (HRQoL) (42) The impact of ePROs on the quality of life and psychological factors is evident in studies by David Riedl et al. (22), Tanja Sprave et al. (23), and Debra A. Patt et al. (24). Riedl et al. (22) explored the ability of adult patients to complete routine ePRO assessments, while Sprave et al. (23) investigated the study of integrating ePROs into the treatment surveillance pathway for Head and Neck Cancer patients. Patt et al. (24) emphasized the significant impact on hospital utilization and cost of care.

Communication and patient engagement have been strengthened by two-way communication channels between patients and caregivers, facilitating compliance tracking, real-time communication with clinicians, and the delivery of self-care instructions targeted to reported symptoms. The ability to effectively communicate with and engage patients is a cornerstone of successful ePRO system implementation (59, 67). David Riedl et al. (22) from Austria highlighted the importance of tailoring ePRO assessments to different age ranges, showcasing the need for personalized communication strategies. Moreover, Data security measures such as data encryption and secure transmission protocols ensure the confidentiality of patient information (28, 34), the integration of Electronic Patient-Reported Outcome (ePRO) systems into oncology care demands a steadfast commitment to data security and encryption. Authors like Silvia Hofer et al. (30) from Switzerland, assessing the usability of the ChemoPRO® app, emphasized the criticality of secure home-based ePRO assessments and electronic data collection while maintaining the confidentiality and privacy of patients’ health-related data. Similarly, Franziska Geese et al. (32) from Switzerland, evaluating the feasibility and acceptability of the eRAPID system, highlighted the need for secure Electronic Patient-Reported Outcome Measures (ePROMs). The assessment of quality of life, functionality, needs, distress, and fear of progression necessitated a robust infrastructure, ensuring the confidentiality of patient-reported information.

Numerous studies found the potential of ePRO systems in advancing research and clinical trials. Gvozdanovic et al. (33) from the United Kingdom leverage Vinehealth, a smartphone application, that uses behavioural science and machine learning for palliative STS treatment. Their work emphasizes the impact of treatment on health-related quality of life (HRQoL) and patient-reported outcomes, showcasing the potential for ePRO systems to contribute valuable data to clinical research. Zhang et al. (34) from China, in the Protecty study, delved into the effectiveness of an ePRO system for prostate cancer care. The digital telemonitoring platform not only monitors health status changes but also identifies risk factors, illustrating the multifaceted role ePROs can play in informing symptom management and supportive care within a clinical trial context.

The ability to visualize and report patient-reported data is crucial for healthcare providers. Authors like Patricia Holch et al. (29) from the United Kingdom, developing a smartphone-based app for prostate cancer patients, emphasize real-time transfer and display of patient responses. Their eRAPID system incorporates graphical and tabular summaries, facilitating efficient communication between patients and clinicians. Furthermore, James Convill et al. (46) from the United Kingdom, examining psychosocial factors affecting ovarian cancer survivors, through an ePRO platform, indicated the significance of collecting and visualizing patient data. By using Electronic Patient-Reported Outcome Measures (ePROMs), they not only gather valuable information on psychosocial factors but also identify patients for referral based on their smoking status.

Furthermore, the usability and user experience of ePRO systems have been optimized with user-friendly interfaces, customizable questionnaires, comprehensive training for research assistants, and compatibility with Electronic Health Record (EHR) integration (5) Usability and user experience are paramount for the successful implementation of ePRO systems. The study by Absolom et al. (5) from the United Kingdom, investigating the health-related quality of life in cancer survivors, places a spotlight on the eRAPID system's online symptom reporting, immediate severity-dependent advice, and real-time monitoring. These features contribute to a positive user experience, fostering patient engagement and adherence. Additionally, Sprave et al. (23) from Germany, assessing the feasibility of integrating ePROs for Head and Neck Cancer patients, highlight the improved reporting of symptom burden and increased patient satisfaction with the App-Controlled Treatment Monitoring and Support (APCOT) trial. The positive user experiences reported in this study pave the way for broader adoption of ePRO systems in oncology care.

Remote monitoring and supportive care are central to the patient-centred solutions provided by ePRO systems. Remote monitoring platforms like Noona provide web-based solutions for remote symptom monitoring, educational content delivery, and immediate automated advice for adverse event management, ultimately empowering patients in their care journey (13, 15, 49). Helissey et al. (31) from Helsinki and France, investigating the feasibility of remote patient monitoring, demonstrate the capabilities of the Cureety platform. Personalized symptom questionnaires, treatment advice, and medical assistance calls enable patients to actively engage in their care from a distance. Similarly, Bobby Daly et al. (45) from the United States, exploring factors associated with the adoption and compliance of ePROMs, reveal insights into InSight Care. Daily ePRO assessments with colour-coded alerts provide a mechanism for tracking symptom fluctuation and anticipating acute care events, providing a valuable avenue for remote supportive care.

The use of Patient-Reported Outcomes (PROs) in cancer care is a transformative aspect explored by various researchers. Andrew Harper et al. (25) in Canada assessed the impact of ePROs on adverse events and the total cost of care for metastatic cancer patients, emphasizing the utility of the ESAS-r tool. Eva Oldenburger et al. (26) from Belgium, through an online survey platform, focused on describing symptom severity among adolescents and young adults, highlighting the diverse applications of ePROs in capturing patient-reported outcomes. These studies highlight the versatility and applicability of PROs in oncology, demonstrating their potential to positively impact patient care. Riedl et al. (40) from Austria examined the impact of ePROs on the health-related quality of life of melanoma patients, highlighting the multifunctional web-based application - The Life App, which integrates rehabilitation interventions and showcases the potential of PROs in shaping comprehensive oncological treatment. Furthermore, Riis et al. (52) from Denmark detailed the development and implementation of integrated care pathways (ICPs) for the electronic collection of patient-reported outcomes (ePROs) in lung cancer patients.

Future research in electronic patient-reported outcome (ePRO) systems in cancer could delve into integrating artificial intelligence and machine learning algorithms for personalized symptom management, alongside wearable devices and mobile health applications for real-time monitoring. Additionally, evaluating the economic impact of ePRO implementation, conducting longitudinal studies on patient outcomes, integrating patient-reported outcomes into clinical trials, cross-cultural validation of ePRO systems, and investigating user experience and stakeholder engagement strategies are crucial. These research endeavours would contribute to enhancing patient-centred oncology care by optimizing treatment strategies, promoting continuous patient engagement, and ensuring the usability and acceptability of electronic solutions across diverse healthcare settings and patient populations.

## 5 Implications

Our study exhibited several strengths. Firstly, it provided a comprehensive analysis of various electronic health systems utilized in electronic patient-reported outcomes in cancer care, highlighting the widespread adoption of systems like CHES, Noona, and EPIC Electronic Health Record (EHR). This detailed examination provides valuable insights into the landscape of electronic systems in oncology care, setting a foundation for future studies to build upon. Secondly, by focusing on ePRO systems and ePROMs, our research sheds light on the pivotal role these technologies play in capturing patient experiences and enhancing patient-centred solutions. This emphasis highlights the importance of leveraging electronic systems to transform cancer care delivery.

## 6 Limitations

Despite its strengths, this review study had several limitations that provide valuable insights for future research. Firstly, due to time constraints, the study focused on published papers from three main databases, suggesting the need for a more extensive survey across diverse sources. Secondly, while ePRO interventions were examined, other technologies like artificial intelligence and wearable devices were not included in this research. Thirdly, the exclusion of various types of papers such as reports and editorials may have limited the scope of analysis. Fourthly, the study did not include systematic review studies, indicating a potential gap in synthesizing existing evidence. Lastly, the restriction to English publications implies a need for future studies to consider a more inclusive approach to analyzing ePRO systems in cancer care.

## 7 Conclusion

In this comprehensive study, we conducted a thorough analysis of electronic systems and platforms utilized in cancer care, focusing on their capabilities, frequencies of use, and contributions to patient-centred outcomes. Our findings indicate the diverse landscape of electronic patient-reported outcome (ePRO) systems, each providing unique functionalities tailored to address specific needs within oncology settings. Among the identified systems, PRO-CTCAE and CHES emerged as the most frequently mentioned platforms, highlighting their significance in capturing patient-reported outcomes and facilitating electronic data capture and interpretation. While these systems play pivotal roles in enhancing patient care and treatment monitoring, other platforms such as eRAPID, Noona, and PROMPT-Care also contribute significantly to patient engagement and symptom management.

Our analysis revealed a diverse range of capabilities within these systems, with Symptom Assessment and Management being the most frequently studied. These capabilities enable early symptom detection, personalized patient communication, and active patient participation in their care, thereby improving treatment outcomes and overall patient experience. Furthermore, our study provides information about the distribution of system types, with web-based platforms being the most prevalent followed by mobile-based and computer-based systems. Understanding the prevalence and functionalities of these systems provides valuable insights for healthcare providers and researchers aiming to implement or optimize electronic solutions in cancer care.

In general, this study enhances our understanding of the electronic systems and platforms available for cancer care, emphasizing their role in improving patient outcomes, enhancing communication between patients and healthcare providers, and facilitating personalized treatment approaches. Moving forward, continued research and innovation in electronic health solutions will be crucial for advancing patient-centred oncology practices and optimizing the delivery of cancer care.

## 8 Summary table

**Table:** 

What is already known on this topic • Various ePRO systems are utilized in cancer care, contributing to patient-centred solutions. • Different capabilities of ePRO systems are explored, with Symptom Assessment and Management being the most studied. • Different types of ePRO platforms are used in cancer care, with Web-based platforms being the most prevalent. • What this study adds • Provides a comprehensive analysis of ePRO systems, highlighting frequent systems like PRO-CTCAE and CHES. • Identifies key capabilities of ePRO systems, showcasing the prevalence of functions like Remote Monitoring and Supportive Care. • Analyzes distribution of ePRO platform types, indicating a preference for Web-based and Mobile-based platforms. • How this study might affect research, practice or policy • Informs the adoption of electronic solutions in cancer care, guiding healthcare providers and researchers. • Guides implementation and optimization of electronic solutions in cancer care, enhancing patient outcomes and communication. • Informs decisions on the adoption and development of electronic systems in oncology, aligning with modern healthcare delivery models.

## Data Availability

The original contributions presented in the study are included in the article/Supplementary Material, further inquiries can be directed to the corresponding author.
