# Supplementary material for: Electronic patient-reported outcome systems and capabilities in cancer care: a systematic review
Source: Front Digit Health. 2025 Aug 18;7:1560533. doi: 10.3389/fdgth.2025.1560533 (PMC12399665; doi:10.3389/fdgth.2025.1560533)
Supplement: Supplementary file 1 [file Datasheet1.docx]

**Appendix A.** Search Strategy

| **Database** | **Search Strategy** |
| --- | --- |
| Scopus | TITLE-ABS-KEY ( ( ( "electronic patient-reported outcome" OR "electronic patient reported outcome" OR "electronic patient-reported outcome measure" OR "electronic patient reported outcome measure" OR "ePRO" OR "electronic Patient-Self Reporting" ) AND ( "neoplasms" OR "Oncology" OR "Cancer" OR "tumor" ) ) ) AND ( LIMIT-TO ( DOCTYPE , "re" ) OR LIMIT-TO ( DOCTYPE , "ar" ) ) AND ( LIMIT-TO ( LANGUAGE , "English" ) ) |
| Web of Science | (("electronic patient-reported outcome" OR "electronic patient reported outcome" OR "electronic patient-reported outcome measure" OR "electronic patient reported outcome measure" OR "ePRO" OR "electronic Patient-Self Reporting") AND ("neoplasms" OR "Oncology" OR "Cancer" OR "tumor")) (Topic) and Review Article or Article (Document Types) and English (Languages) |
| PubMed | (("electronic patient-reported outcome"[Title/Abstract] OR "electronic patient reported outcome" OR "electronic patient-reported outcome measure"[Title/Abstract] OR "electronic patient reported outcome measure"[Title/Abstract] OR "ePRO"[Title/Abstract] OR "electronic Patient-Self Reporting"[Title/Abstract]) AND ("neoplasms"[Title/Abstract] OR "Oncology"[Title/Abstract] OR "Cancer"[Title/Abstract] OR "Tumor"[Title/Abstract])) Filter: English |
