# Supplementary material for: Electronic patient-reported outcome systems and capabilities in cancer care: a systematic review
Source: Front Digit Health. 2025 Aug 18;7:1560533. doi: 10.3389/fdgth.2025.1560533 (PMC12399665; doi:10.3389/fdgth.2025.1560533)
Supplement: Supplementary file 2 [file Datasheet2.docx]

**Appendix B.** Summary characteristics of articles included

| **Number** | **Author** | **Year** | **Country/State** | **Objective** | **Participants** | **Cancer Type** | **System Name** | **System Capabilities** | **Type of Platform** |
| --- | --- | --- | --- | --- | --- | --- | --- | --- | --- |
|  | Natalie R Dickson, et al ^14^ | 2024 | USA | To evaluate the utilization and clinical impact of an electronic patient-reported outcome (ePRO) tool in patients with solid tumors undergoing immuno-oncology (IO) therapy | 538 patients in the historical control (HC) cohort, 1014 patients in the ePRO cohort, with 319 ePRO users and 695 non-users | Solid tumors (non-small cell lung cancer [NSCLC], melanoma, renal cell carcinoma, bladder cancer, head and neck cancer) | Noona® patient outcomes management solution (Varian Medical Systems) | Capture patient-reported outcomes (PROs) online, integration with electronic medical records (EMRs), delivery of PRO-CTCAE questionnaires, symptom tracking and management, real-time alerts for healthcare providers | Web- application |
|  | David Riedl, et al ^22^ | 2023 | Austria | To investigate the ability of adult patients of different age ranges to complete routine ePRO assessments and to identify factors associated with completion and the need for assistance. | 5571 patients (mean age: 60.3 years, range 18 to 93 years) in Inpatient Rehabilitation setting | Various cancer types (breast, hemoblastoses, prostate, uterine/ovarian, colon, head/neck, lung, stomach, rectum) | Computer-based Health Evaluation System (CHES) | Remote completion and in-house assessments, Different completion rates for various cancer types | Computer-based |
|  | Tanja Sprave, et al ^87^ | 2023 | Germany | -Investigate the feasibility of integrating electronic patient-reported outcomes (ePROs) in the treatment surveillance pathway for HNC patients during radiotherapy.  -Assess the impact of app-based ePRO monitoring on global and disease-specific quality of life and patient satisfaction. | 100 enrolled, 93 evaluable | Head and neck cancer (HNC)  (Oropharynx,  Larynx,  Hypopharynx,  Nasopharynx, and  Parotid glands) | App-Controlled Treatment Monitoring and Support for Patients With Head and Neck Cancer (APCOT) Trial | Provided daily app-based ePRO surveillance, improved reporting of symptom burden, and increased patient satisfaction. | Mobile-based application |
|  | Debra A. Patt, et al ^23^ | 2023 | United States | To evaluate the impact of electronic patient-reported outcomes (ePROs) on adverse events and total cost of care among patients with metastatic cancer enrolled in the Centers for Medicare & Medicaid Services' Oncology Care Model (OCM) program | Initially, 1,630 patients with cancer; 831 met the selection criteria, 458 matched patients were identified | Metastatic Breast,  Chronic leukaemia,  Lung,  Lymphoma,  Multiple myeloma,  Prostate, and  Small intestine/colorectal cancer | Electronic Patient-Reported Outcomes (ePRO) system | Feasibility of implementing ePRO, significant impact on hospital utilization and cost of care  Symptom monitoring with ePROs improved the quality and value of cancer care  delivery by reducing hospitalizations, emergency visits, and deaths while  lowering the cost of care in a large oncology practice | N/A |
|  | Andrew Harper, et al ^24^ | 2023 | Canada | 1. Describe symptom severity among adolescents and young adults (AYA) with cancer at diagnosis and 1 year after diagnosis. 2. Identify demographic and clinical risk factors for higher symptom severity.  3. Evaluate symptom trajectories among AYA with cancer during the year following diagnosis.  4. Compare symptom severity and trajectories with older adult patients with cancer. | 937 adolescents and young adults; 473 at diagnosis, 322 at 1 year after diagnosis | Various cancer types, including Breast, Central nervous system, Endocrine, Gastrointestinal, Genitourinary, Gynecologic, Head and neck, Hematologic, Intrathoracic, Melanoma, Sarcoma, etc. | Patient-Reported Outcomes in Oncology in Alberta | - Utilizes the ESAS-r tool for assessing symptom severity. - Electronic collection of patient-reported outcomes data across 17 ambulatory cancer centres in Alberta. | Web-based |
|  | Eva Oldenburger, et al ^78^ | 2023 | Belgium | Explore the opinions of healthcare providers (HCP) active in radiation oncology in Belgium on using ePROMs for symptom follow-up after palliative radiotherapy. | 128 respondents, including Radiation Oncologists, Nurses, Radiation Therapy Technologists, Clinical Support Managers, and Quality Managers. | N/A | Online survey platform (SurveyMonkey) | N/A | Web-based (Email) |
|  | Williams LA, et al. ^16^ | 2023 | USA | To rapidly develop, launch through an electronic patient portal, and provide initial validation for a PRO measure of COVID-19 symptom burden in patients with cancer. | 600 participants diagnosed with both cancer and COVID-19 | Various types of cancer in individuals also diagnosed with COVID-19 | -EPIC Electronic Health Record (EHR) System | Efficient participant recruitment, and seamless data collection through the EPIC EHR system | Web-based electronic patient portal |
|  | Warnecke E, et al. ^11^ | 2023 | Germany | To compare the information provided by ePROMs and nurse-reported assessments in order to identify overlaps and differences in the assessment of current symptom burden among oncological inpatients. | 230 inpatients | Soft-tissue sarcoma, Lung, Uveal melanoma, Gastrointestinal, Hepatobiliary and pancreatic cancer | Electronic Psycho-Oncological and Palliative Screening (ePOS) | - Enhanced assessment of psychological distress  - Early symptom detection and management  - Improved patient-provider communication | Computer Based |
|  | Moradian S, et al ^19^ | 2023 | Canada | To develop an eHealth platform for cancer patients to manage symptoms and interact with healthcare professionals. | N/A | Various types of cancer in individuals | V-Care Platform | Patient Terminals:  -Access irAE history  -Search cancer info  -Learn to manage irAEs  -Prioritize user-friendly interface  Clinician Terminals:  -Securely access patient data  -Monitor e-PROs  -Communicate with patients  -Enhance patient care  Clinical Server:  -Centralized data management  -Stores patient info  -Generates alerts  -Supports decision-making  -Ensures privacy and security  -Facilitates data exchange  -Collects e-PROs and generates alerts  -Automates processes for severe issues  Researcher Terminals:  -Access and analyze anonymized patient data for research  Other:  -Real-time symptom monitoring  -Communication with clinicians  -Data exchange  -Alert generation | Mobile App |
|  | Mohseni M, et al. ^88^ | 2023 | Iran | Develop a smartphone-based app for electronic reporting of outcomes by patients with prostate cancer | Specialists (n=15), Patients (n=21) | Prostate cancer | Prostate cancer application | -Facilitating reporting of outcomes and side effects and improving patient care.  - Improving communication  -Facilitate messaging between patients and physicians  -Evaluating quality of life | Mobile App |
|  | McMullan C, et al. ^18^ | 2023 | United Kingdom | To assess the usability of the ChemoPRO® app among people with lived experience of cancer. | 10 participants with lived experience of cancer. | Leukaemia, Breast, Multiple, Myeloma, Stomach, Bowel, Rectal, Sarcoma | ChemoPRO® | ChemoPRO® is linked to the ChemoCare® platform and includes features for:  -Recording symptoms,  -Managing appointments, and  -Providing information to patients. | Mobile app |
|  | Bojan Macanovic et al. ^25^ | 2023 | Ireland | To investigate the feasibility of implementing a remote patient monitoring system using an electronic patient-reported outcomes (ePROs) platform in a tertiary cancer centre in the Republic of Ireland. | 13 patients and 5 staff | Breast  Melanoma  Colorectal  Lung | ONCOpatient® | Symptom assessment, medication management, communication, alerts, compliance monitoring, data encryption, mobile-based. | Mobile App |
|  | Patricia Holch, et al.^64^ | 2023 | United Kingdom | Establish feasibility and acceptability of the eRAPID system | 167 (73.2% consented and randomized) | Prostate, lower gastrointestinal, and gynaecological cancers | eRAPID (Electronic patient self-Reporting of Adverse-events: Patient Information and aDvice | -Real-time transfer and display of patient responses  -Clinical alerts for severe symptoms  *eRAPID Website Portal Screen:*  -Provides a user-friendly interface for patients to access the ePRO system.  *QTool Patient Welcome Page:*  -Displays links to questionnaires, previous responses, and feedback; serves as a central hub for patient interaction with the ePRO system.  -Serves as a central hub for patient interaction with the ePRO system.  *eRAPID Symptom Report:*  - Provides an example of the eRAPID symptom report to give patients insights into their reported symptoms.  *AE Self-Management Advice:*  -Generated from QTool when patients report mild/moderate symptoms.  *Patient Direction for Low-Level AE:*  -Guides patients to the eRAPID website for self-management advice when reporting low-level adverse events.  *Severe AE Reporting Advice:*  -Generates advice (in red) for patients to telephone the hospital if they report a severe adverse event.  *Tabular Summary of AE Reported:*  -Presents patients with a tabular summary of their reported adverse events.  *Graphical Summary of Responses:*  -Provides patients with a graphical representation of their responses over time.  Graphical Display of One-Time PRO Results:  - Provides clinicians a graphical representation of patient-reported outcomes from a single assessment.  *Tabular Display of PRO Reported Results in EPR:*  -Displays patient-reported results in the Electronic Patient Record (EPR) in tabular form.  -Highlights severe symptoms in red for quick identification by clinicians.  *Graphical Display of Completion Over Time:*  -Shows clinicians a graphical representation of patient completion over time, with red triangles indicating chemotherapy cycles. | Web-based |
|  | Silvia Hofer et al.^89^ | 2023 | Switzerland | To assess the impact of treatment on health-related quality of life (HRQoL) and patient-reported outcomes in palliative STS treatment. | The study was terminated early due to the COVID-19 pandemic, and only 11 patients were randomized and 10 evaluated. | Soft Tissue Sarcoma (STS) | Digital Health Management from Compliance Solutions GmbH | - Home-based ePRO assessments,  - electronic data collection, assessment of HRQoL and  - Treatment satisfaction. | Mobile- based |
|  | Helissey. et al^90^ | 2023 | Helsinki and  France | Effectiveness of electronic patient reporting outcomes, by a digital telemonitoring platform, for prostate cancer care: the Protecty study | 61 patients | Prostate cancer | Cureety | *Personalized Symptom Questionnaires:*  - Enables patients to complete personalized symptom questionnaires.  - Classifies patients into different health states based on their responses.  *Treatment Advice*:  - Provides personalized treatment advice based on the reported symptoms and health states.  *Medical Assistance Call*  - Allows patients to initiate a call for medical assistance if needed. | Mobile Application |
|  | Franziska Geese, et al ^96^ | 2023 | Switzerland | Explore the potential of ePROMs in clinical practice for assessing the quality of life, functionality, needs, fear of progression, distress, and care quality in sarcoma centres | 55 patients from three sarcoma centres | Sarcoma | Electronic Patient-Reported Outcome Measures (ePROMs) | Evaluate quality of life, functionality, needs, distress, fear of progression | N/A |
|  | Andrew Gvozdanovic, et al ^26^ | 2022 | United Kingdom | To assess the feasibility of Vinehealth integration into brain tumour care | Six patients were initially recruited, and four engaged with the Vinehealth application throughout the study period. | Brain tumours include glioblastoma, metastasis from triple-negative breast carcinoma, and haemangioblastoma. | Vinehealth - a smartphone application using behavioural science and machine learning for cancer patients’ self-management and psychological well-being. | Data collection (symptoms, activity, medication, well-being), delivery of educational content, subjective care improvement. | Mobile-based application |
|  | Yening Zhang et all^27^ | 2022 | China | To track patient-reported health status changes over time in Chinese advanced cancer patients and explore the risk factors affecting their health status. | 103 patients completed a baseline survey (T = 0) and two follow-up surveys (T1 = 14 days, T2 = 28 days). | Advanced stages of cancers, including Stage III without curative treatment chance and Stage IV)  Lung, gastric, oesophageal, liver, colorectal, and breast cancer | ePRO system (Electronic Patient-Reported Outcome system) | Monitoring health status changes, identifying risk factors, and informing symptom management and supportive care. | Mobile phone |
|  | Liyan Zhang, et al^28^ | 2022 | China | To compare the efficiency between electronic patient-reported outcomes (ePRO) and traditional follow-up models in cancer immunotherapy. | 278 patients (141 in the intervention group, 137 in the control group) | Gastric,  Esophageal,  Lung,  Pancreatic,  Colorectal, Breast,  Brain,  Liver,  Kidney, and  others | Electronic patient-reported outcome (ePRO) follow-up system | - Weekly symptom monitoring  - Image recognition for irAE evaluation  - Automatic standardized advice for mild irAEs  - Immediate alert for severe irAEs  - 6-month follow-up period or until treatment completion | Computer-based |
|  | Wickline, et al ^70^ | 2022 | United States | Usability and acceptability of the electronic self-assessment and care (eSAC) program in advanced ovarian cancer | Total Sample (N = 134); Device Interview Sample (n = 18); Usability Interview Sample (n = 19) in Ambulatory Setting: | Advanced ovarian cancer | Electronic self-assessment and care (eSAC) | Patient self-reporting of symptoms, quality of life measures, and decision-making preferences during cancer therapy. Delivery of self-care instructions targeted to reports of moderate-severe symptoms. Real-time summaries and alerts for clinicians. | Web-based |
|  | Tolstrup,et al^76^ | 2022 | Denmark | -To examine the impact of using electronic patient-reported outcomes (ePRO) with triggered alerts as an add-on to standard care on the health-related quality of life (HRQoL) of melanoma patients receiving checkpoint inhibitors.  -To investigate the association between immune-related adverse events (irAEs) severity and HRQoL. | Patients (N=138) | Melanoma | ePRO system (software platform AmbuFlex) | The ePRO system included triggered alerts for symptoms and clinician feedback during clinical encounters. It allowed patients to report symptoms electronically. | Web-based |
|  | Tang et al ^29^ | 2022 | China | To describe the implementation process and evaluation of an ePRO platform for symptom management in cancer patients, share experiences, and assess feasibility, safety, and efficacy. | A total of 161 patients with advanced cancer were enrolled in the study, although completion rates varied across the seven follow-up assessments. | Patients with advanced cancer, including lung, liver, gastric, oesophagal, colorectal, and breast cancer. | The study introduced three components of the ePRO system: ePROhub, ePRO-Doctor Client, and ePRO-Patient Client. EPROhub served as the primary data collection and management system, while the ePRO-Doctor Client allowed healthcare providers to monitor patient progress and receive alerts. The ePRO-Patient Client was designed for patients to conveniently report their symptoms. | -Integration with WeChat, a popular social app, for patient reporting and convenience.  - Real-time data collection and monitoring of symptoms  - Implementation of symptom-tracking promotion strategies, including automated reminders and alerts.  - Comprehensive training and evaluation for research assistants to ensure smooth operation of the system. | Web-based |
|  | Gabrielle B. Rocque, et al ^30^ | 2022 | United States | To adopt a remote symptom monitoring intervention developed in research settings for implementation in real-world clinical settings at two cancer centres. | Phase I: 23 patients; Phase II: 35 patients (Myeloma and Acute Leukemia) | Lymphoma, Breast, Gastrointestinal, Genitourinary, Myeloma, Acute Leukemia | Carevive | - Remote symptom assessment. - Automated alerts. - Integration with EHR (Electronic Health Record).  - Patient education.  - Real-time survey monitoring. | Software |
|  | David Riedl et al ^67^ | 2022 | Austria | To assess the impact of multidisciplinary inpatient rehabilitation on the health-related quality of life (HRQOL) and physical fitness of pediatric cancer survivors. | 236 pediatric cancer survivors aged 5-21 years and 478 parents (as proxy respondents). | leukemias, lymphomas,  Central Nervous System (CNS) tumours Brain, Bone, Soft tissue, Blood, immune system and others. | The Life App is a multifunctional web-based application for rehabilitation management. | The rehabilitation interventions provided included physiotherapy, psychotherapy, nutrition counselling, and other multidisciplinary approaches. | Web-based application |
|  | Nordhausen, et al ^4^ | 2022 | Germany | To evaluate the implementation of electronic patient-reported outcomes (e-PRO) in inpatient radiation oncology | The study involved a total of 568 patients. | Patients with various cancer | Computer-based Health Evaluation System (CHES) | - CHES was used for collecting, scoring, and presenting e-PRO results in electronic patient records.  - Patients could complete assessments on tablets or individual devices. - Real-time graphic displays of e-PRO results were available. | Computer-based |
|  | Mangyeong Lee et al ^31^ | 2022 | Korea | To identify factors associated with the adoption and compliance of electronic patient-reported outcome measure (ePROM) among cancer patients in a real-world setting | 580 cancer patients | Various cancers (e.g., breast, lung, gastric, colorectal, lymphoma, head and neck, others) | ePROM mobile application | - Ease of reporting symptoms via smartphones - Data visualization for symptom change over time - Access to information for self-management | Mobile application |
|  | Fay J. Hlubocky, PhD, MA, et al. ^79^ | 2022 | United States | To examine the prevalence of psychosocial factors affecting quality of life in ovarian cancer survivors using an electronic patient-reported outcome (ePRO) platform | 174 out of 300 ovarian cancer survivors | Ovarian cancer | ePRO platform | Assessment of psychosocial factors, distress, post-traumatic growth, resilience, and financial stress affecting HRQOL. | Web-based |
|  | Joachim Graf, et al. ^32^ | 2022 | Germany | To analyze the acceptance and evaluation of a tablet-based ePRO app for breast cancer patients and examine its suitability, effort, and difficulty in the context of HRQoL and sociodemographic factors. | 106 women with adjuvant or advanced breast cancer at 2 major university hospitals in Germany. | Breast Cancer | PiiA (patient interactively informs doctor) | Efficiently collect HRQoL data, high usability ratings, suitable for breast cancer patients. | Web-based |
|  | Afaf Girgis, et al ^56^ | 2022 | Australia | To evaluate the processes and success of implementing the PRM system in the routine care of patients diagnosed with lung cancer. | 48 patients diagnosed with lung cancer completed 90 assessments during the 5-month implementation period. | Lung cancer | PROMPT-Care: PRM system (Patient Reported Outcome Measures for Personalized Treatment and Care) | The PRM system is fully integrated into the patient's electronic medical records and supports patient management through various assessments, automated clinical alerts, and tailored self-management resources. | Web-based |
|  | Bobby Daly, et al ^33^ | 2022 | United States | Assess the clinical value of daily electronic patient-reported outcomes (ePROs) for cancer patients undergoing antineoplastic treatment | 217 patients (median age 66, 103 women, and 114 men) | Breast,  head and neck,  gastrointestinal,  genitourinary,  gynaecology,  lymphoma,  melanoma,  thoracic, and  soft cancers | InSight Care | - Daily ePRO assessments with red and yellow alerts  - Symptom fluctuation tracking over a week  - Association of alerts with future acute care events | Mobile-based |
|  | James Convill, et al. ^57^ | 2022 | United Kingdom | - Investigate the level of agreement between clinician-reported and self-reported patient smoking status during the first visit to a cancer centre.  - Examine the self-reported frequency of smoking cessation after the diagnosis of lung cancer. | 195 patients were included in the primary analysis. | Lung cancer | Electronic Patient-Reported Outcome Measures (ePROMs) | - Collecting patient data on smoking status. - Identifying patients for smoking cessation referral. | N/A |
|  | S. Boeke, et al ^34^ | 2022 | Germany | To assess patient acceptance of physical activity (PA) monitoring in an outpatient setting during radiotherapy and to correlate changes in PA with toxicity and changes in quality of life (QoL). | 23 patients | Breast  Head and Neck, Lung  Anal, Esophageal, and  Pancreatic cancer | Clinical trials: GIROfit Phase 2 Pilot Trial | Monitoring physical activity, identifying patients in need of supportive care, and potential for remote monitoring | N/A |
|  | Adeola Bamgboje‐Ayodele, et al ^58^ | 2022 | Australia | To detail the development and implementation of integrated care pathways (ICPs) for electronic collection of patient-reported outcomes (ePROs) in lung cancer patients in oncology settings | 96 staff members participated in engagement activities across three hospitals. | Lung Cancer | PROMPT-Care (Patient Reported Outcome Measures for Personalized Treatment and Care) | Adaptation of ICP through engagement, integration of ePROs, decision-making for patient cohorts, onboarding processes, assessment screening, clinical alerts management, and referral pathways | Web-based |
|  | Laura Takala, et al ^35^ | 2020 | Finland | Assess the usefulness of electronic patient-reported outcomes (ePROs) during adjuvant radiotherapy (RT) in patients with early breast cancer. | 253 patients with breast cancer receiving RT | Breast cancer | Noona (Varian Medical Systems, Inc., Palo Alto, CA) for  Cancer Follow-Up Application (CFUA) | Two-way communication between patients and caregivers, compliance and active participation, and collection of real-world data on patient symptoms during treatment. | Web-mediated application |
|  | Olga Strachna, et al ^91^ | 2021 | United States | To develop an electronic PROs (ePROs) program for head and neck cancer patients and evaluate its feasibility and impact. | 4,154 patients | Head and neck cancer | Head and Neck PROs Oncology platform | - Longitudinal assessment of treatment-related side effects - Data visualization for monitoring | Mobile-based |
|  | Dag Rune Stormoenet al ^73^ | 2021 | Denmark | To describe Patient-Reported Outcomes (PROs) from patients with metastatic castration-resistant prostate cancer (mCRPC) receiving oncological treatment and compare them with adverse events from registration studies | 54 patients with mCRPC receiving medical oncological treatment | Metastatic castration-resistant prostate cancer (mCRPC) | PRO-CTCAE (Patient Reported Outcome of Common Terminology Criteria for Adverse Events) | PRO-CTCAE assesses the existence, frequency, and severity of symptomatic toxicities, with a focus on symptoms interfering with usual or daily activities. | Web-based |
|  | Cathrine Lundgaard Riis, et al ^36^ | 2021 | Denmark | To examine the impact on service use, workflow, and workload after introducing ePRO-based individual follow-up for early breast cancer treatment. | Initially, 129 women were assessed, 64 in SFU, and 60 in PIFU; the final assessment included 47 participants for PREMs. | Early-stage breast cancer | ePRO-based individual follow-up (PIFU) | Utilization of ePROs for patient triage, personalized care allocation, reduction in unnecessary consultations, patient empowerment | N/A |
|  | Sissel Ravn, et al ^71^ | 2021 | Denmark | To evaluate the effect of a follow-up supported by electronic patient-reported outcomes (ePRO) on Patient Activation (PA) and Patient Involvement (PI) in patients undergoing intended curative complex surgery for advanced cancer. | 187 patients who had undergone intended curative complex surgery for advanced cancer at two different departments at Aarhus University Hospital. | Patients with metastases to the peritoneal surface undergoing intended curative complex surgery for advanced cancer. | ePRO (Electronic Patient-Reported Outcomes) | ePRO was designed to collect patient-reported outcomes data, visualize patient responses to clinicians, and facilitate patient involvement in follow-up consultations. | N/A |
|  | Maria Kristiina Peltola, et all ^13^ | 2021 | Finland | To assess the suitability of the Noona ePRO application for patients with cancer, nurses, and doctors at Helsinki University Hospital | - Patients: 44 - Health care professionals: 17 | Various solid tumour types | Noona | - Noona is a web-mediated application that allows patients to report symptoms and adverse events from home via a computer or a smart device (e.g., smartphone, tablet). - It supports communication between patients and healthcare professionals, facilitating remote monitoring of patients with cancer. - Noona is intended for use during cancer treatment phases and follow-up and rehabilitation periods. - Patients can report symptoms using question wizards that cover clinically relevant questions, such as pain, fatigue, nausea, vomiting, and bowel symptoms. | Web- application |
|  | Debra Patt, et al ^37^ | 2021 | United States | To determine the feasibility of real-world implementation of electronic patient-reported outcomes (ePROs) among patients with cancer at a large community oncology practice. | 4,375 patients | Breast cancer,  Chronic leukaemia,  Lung cancer,  Lymphoma,  Multiple myeloma. and  Prostate cancer, Small intestine/colorectal cancer | ePRO  system:  Navigating Cancer ePRO platform, Health Tracker | - Real-time symptom reporting  - Immediate care for high-risk patients  - Data stratification by risk level  - Data visualization for care coordination  - Tracking of symptoms over time | Web-based |
|  | Thomas Licht, et al ^38^ | 2021 | Austria | Investigate cancer survivors' health-related quality of life (HRQOL), specific deficiencies related to underlying disease or treatment, and benefits of rehabilitation in a variety of cancer entities. | 4,401 cancer survivors | Various cancer entities, including head and neck,  esophageal,  gastric,  colon,  rectal,  liver,  pancreatic,  lung,  skin,  breast,  uterine,  ovarian,  prostate,  testicular,  renal,  bladder,  brain,  thyroid,  malignant lymphomas,  multiple myeloma,  leukaemias, and other cancer types | Computer-Based Health Evaluation Software (CHES) for data gathering and analysis. | The rehabilitation program includes physical, psycho-oncological, and educative components, such as lifestyle modification and nutrition. | Computer-Based (Software) |
|  | Jeeyeon Lee, et al ^39^ | 2021 | South Korea | To evaluate the degree of depression and anxiety in patients with breast cancer during the treatment period and short-term follow-up. | 137 patients with breast cancer | Breast cancer | SAAD (Serial Assessment of Anxiety and Depressive Symptoms in Breast Cancer) | Web-based platform for depression and anxiety assessment. | Web-based |
|  | Kaitlyn Lapen, et al ^40^ | 2021 | United States | Develop and study the implementation of a remote system for toxicity assessment and management of acute breast radiation side effects using electronic patient-reported outcomes (ePROs) | 678 patients | Breast cancer | PRO-CTCAE | - Remote symptom monitoring for patients undergoing radiation therapy - Weekly ePRO assessment distributed during and immediately after radiation | Web-based |
|  | M. Kay M. Judge, et al ^65^ | 2021 | United States | To identify implementation issues and evaluate the efficacy of an electronic patient self-reporting pain device in community-based cancer clinics. | 178 cancer patients (33 in the pilot phase and 145 in the RCT phase) in community-based clinics | Various types of Cancer patients | PAINReportIt® | - Measures multiple dimensions of pain - Provides an easy-to-read summary for both patients and clinicians - Time-efficient for patients and clinicians - Potential to improve pain management and documentation | Web-Based Mobile App (Interact App) |
|  | Olga Generalova, et al ^15^ | 2021 | United States | Feasibility, implementation, and healthcare utilization outcomes of an electronic PRO (ePRO) application for cancer patients at an academic medical centre. | 72 patients | Patients with advanced cancer in the thoracic, gastrointestinal, and genitourinary oncology groups | The Noona platform \| | - Ability to capture PROs in oncology.  - Compatibility with EHR integration.  - Adaptability to institutional preferences.  - Inclusion of oncology-specific modules.  - Ability to prompt patients to contact their medical team immediately for severe symptoms.  - Integration of symptom data into clinical workflows. | Mobile-based |
|  | Jim W. Doolin, et al ^80^ | 2021 | United States | To assess the feasibility of implementing an electronic patient-reported outcomes (ePRO) system for patients starting oral chemotherapy at a cancer centre improving patient monitoring, and symptom assessment. | 62 patients who started a new oral chemotherapy regimen agreed to receive online ePROs, and 25 of them completed the ePRO (40% completion rate). A historical cohort of 50 patients was also used for comparison. | N.A | REDCap electronic data capture tools | The ePRO system allowed patients to report treatment-related concerns and symptoms. Responses triggered clinical outreach for problem management. | Web-Based |
|  | Kate Absolom, et al ^41^ | 2021 | United Kingdom | To evaluate the impact of eRAPID on symptom control, healthcare use, patient self-efficacy, and quality of life in a patient population predominantly treated with curative intent during chemotherapy | 508 consenting patients and 55 health professionals | Colorectal, breast, or gynaecological cancers | eRAPID (Electronic patient self-reporting of adverse events: Patient Information and aDvice) | 1. Online symptom reporting. 2. Immediate severity-dependent advice. 3. Integration with electronic patient records (EPRs). 4. Real-time monitoring. | Web-based |
|  | Dylan M. Zylla, et al ^42^ | 2020 | United States | To assess the feasibility of using electronic patient-reported outcomes (ePROs) for symptom monitoring in patients with advanced cancer | 80 patients with stage IV non-hematologic malignancies on chemotherapy | Stage IV non-hematologic malignancies include lung, colorectal, prostate, pancreas, head and neck, oesophagal and stomach, breast, ovarian, cervical, endometrial, liver/bile duct, and kidney cancers. | Epic MyChart | - Efficiently collects and integrates patient-reported symptoms into the electronic health record. - Facilitates timely communication with clinicians and patients. - Provides automated alerts for concerning symptoms. - Tracks patient-reported symptoms in real-time. - Allows better symptom management and care planning. | Computer- Web-based |
|  | Christine Tran, et al ^92^ | 2020 | United States | To explore the feasibility and acceptability of collecting electronic patient-reported outcomes (ePROs) using validated health-related quality of life (HRQoL) questionnaires for prostate cancer. | 29 patients in total; 1 patient excluded from analysis | Prostate cancer | Strength Through Insight | Collecting ePROs using validated HRQoL questionnaires, facilitating communication between patients and caregivers, monitoring patient outcomes, assessing patients' experiences and needs | Mobile app  Mobile-based |
|  | Lærke K. Tolstrup, et al ^72^ | 2020 | Denmark | Assess the electronic tool's impact on reducing severe adverse events by 50% in melanoma patients undergoing immunotherapy. | 146 melanoma patients participated in the study. | Metastatic melanoma patients receiving immunotherapy. | PRO-CTCAE item library | Active reporting of symptoms | Web-based |
|  | Sahil Sandhu, et al ^43^ | 2020 | United States | To gain insights into oncologists' perspectives regarding the incorporation of electronic patient-reported outcomes (ePROs) into routine cancer care at an academic centre | 16 oncologists with diverse subspecialties and experience in various cancer types | Genitourinary,  Breast,  GI,  Sarcoma,  Urologic,  Thoracic, | The article does not specify the name of the ePRO system used. | - Customizability of ePRO questionnaires - Data visualization for effective information management - User-friendly design for ease of navigation - Automation for streamlined processes  ~~-~~Efficient and standardized documentation | N/A |
|  | Cathrine L. Riis, et al ^44^ | 2020 | Denmark | Evaluate patients' satisfaction with care provided using electronic patient-reported outcomes (ePROs) to individualize follow-up care for women with early breast cancer receiving adjuvant endocrine therapy | 134 women | Early breast cancer | ePRO-based individualized follow-up care | Screening tool for assessing patient problems and individual requirements, Dialogue tool to map patient symptoms and concerns | Computer-based |
|  | H. S. Richards, et al ^45^ | 2020 | United Kingdom | To evaluate the feasibility of a real-time electronic symptom monitoring system for patients after discharge following cancer-related upper gastrointestinal surgery | 40 participants in the study | Cancer-related upper gastrointestinal surgery (oesophageal, gastric, hepato-pancreato biliary cancer) | ePRO surgery system  (Part of eRAPID project) | - Real-time symptom monitoring - Severity-specific tailored self-management advice - Alerts for clinicians based on symptom severity | Web application |
|  | Florence D Mowlem, et al ^46^ | 2020 | United Kingdom | To understand the impact of anticancer treatment on oncology patients' ability to use electronic solutions for completing patient-reported outcomes (ePRO). | Seven individuals with cancer diagnosis and treatment experience. | Breast, Prostate, and Colon/bowel | Signant Health's eCOA software solutions on tablet and mobile devices. | - Electronic Capture of Patient-Reported Outcome Measures  - Employs electronic tools for patients to report symptoms, health-related quality of life, and pertinent data during oncology clinical trials. | Tablet and Mobile app |
| 1. 184 | Christina karamanidoua, et al ^93^ | 2020 | Greece | To develop a novel ePRO-based palliative care intervention for cancer patients by eliciting end-user's needs, judgments of the MyPal system, and recommendations for improvement. | Nine patients with Chronic Lymphocytic Leukemia (CLL) | Chronic Lymphocytic Leukemia (CLL) and Myelodysplastic Syndromes (MDS) | MyPal (https://mypal-project.eu/) | MyPal supports direct contact with healthcare professionals, symptom reporting, and personalized information, and provides specialized data for different patient profiles. | Mobile-based |
|  | Doris Howell, et al ^59^ | 2020 | Canada | To implement electronic Patient Reported Outcomes (e-PROs) in 'real-world' oncology practices for personalized management of generic and targeted symptoms of pain, fatigue, and emotional distress (depression, anxiety). | Over 6000 patients completed e-PROs | Lung and sarcoma cancer | iPEHOC  (Improving Patient Experience and Health Outcomes Collaborative) | Integration of e-PRO data for personalized patient management, improved patient experience, and reduced anxiety and healthcare utilization | Web-based |
|  | Afaf Girgis, et al ^75^ | 2020 | Australia | Evaluate the effectiveness of the PROMPT-Care web-based system in a diverse population of cancer patients by reducing emergency department presentations and other health service outcomes. | 328 patients received the intervention, and 1312 patients were matched as controls. | Patients with solid tumors | PROMPT-Care (Patient Reported Outcome Measures for Personalized Treatment and Care) | -Providing real-time feedback to care providers and patients  - Generating clinical alerts based on predefined thresholds, and  - Providing patient self  - Management resources | Web-based |
|  | Emilie A. C. Dronkers, et al ^97^ | 2020 | Netherlands | To evaluate the implementation of an electronic patient-reported outcome measures (ePROs) system, in the routine care of head and neck cancer (HNC) patients. | -Quantitative: HM group (45 patients), Standard care group (46 patients) - Qualitative: Interviews with 15 HM patients | Head and neck cancer | Healthcare Monitor (HM):  ePRO-based clinical support system | - Monitors physical and psychosocial functioning.  - Provides real-time access to results. - Facilitates individual feedback to patients.  - Allows comparison of individual patient results with peer groups.  - Enhances patient empowerment. | N/A |
|  | Noa Biran, et al ^94^ | 2020 | United States | Evaluate the acceptability and appropriateness of an electronic patient-reported outcome (ePRO) intervention for patients with relapsed and refractory multiple myeloma (RRMM) and explore its impact on clinic workflow | 11 patients with RRMM were recruited, and 9 patients completed the study | Relapsed and Refractory Multiple Myeloma (RRMM) | Medocity Home Health app | The system facilitated the reporting of 17 RRMM symptoms, generated alerts for the clinic when predefined thresholds were met, provided self-management guidance to patients, and allowed for ad hoc symptom reporting. It also had the potential to generate symptom graphs and reports | Mobile App |
|  | Lorraine Warrington, et al ^7^ | 2019 | United Kingdom | To field test the eRAPID system, an online tool for monitoring and managing adverse events in patients with cancer during treatment. | 12 patients receiving chemotherapy for early breast cancer and 10 health professionals (oncologists and specialist nurses). | Patients with early breast cancer were tested in the field usability study. The eRAPID system is being evaluated in a larger population, including patients with breast, gynaecology, or colorectal cancer. | electronic patient self-reporting of adverse events: Patient Information and aDvice (eRAPID) | - Allows patients to complete symptom reports from home. - Provides severity-based self-management advice. - Sends notifications to contact the hospital for severe symptoms. - Patient data is available in electronic records for staff to review. - Includes a graphing feature for patients to review personal symptom data over time. | Web-based |
|  | Hilde Krogstad et al.^47^ | 2019 | Norway | To evaluate the usability of the EirV3 system used for patient-reported outcome measures (PROMs) in cancer care | 37 patients, 17 physicians | Breast, Gastrointestinal, Lymphomas, Prostate, Gynecological, Lung, Malignant melanoma, and Testicular cancers | EirV3 | Patient-centred system with real-time symptom tracking | Computer-based |
|  | Yuichiro Kikawa, et al ^48^ | 2019 | Japan | Evaluation of health-related quality of life (HRQOL) monitoring from home among metastatic breast cancer (MBC) patients using the Computer-Based Health Evaluation System (CHES) | 16 MBC patients who received outpatient chemotherapy or endocrine therapy, both with and without targeted therapy. | Metastatic breast cancer (MBC) | Computer-Based Health Evaluation System (CHES) | CHES electronically collects and stores patient questionnaires for HRQOL assessment. The data can be accessed by medical staff to monitor and display longitudinal data. | Computer-based |
|  | Sanna Iivanainen, et al ^66^ | 2019 | Finland | Investigate whether symptoms collected by the Kaiku Health ePRO tool on cancer patients receiving immune checkpoint inhibitors (ICI) | 37 patients | Various types of cancer | Kaiku Health IO module | *Timely and Continuous Symptom Collection*  - Facilitates ongoing and timely collection of symptoms.  *Self-Management Support (SMS)*  - Provides patients with tools and resources for effective self-management.  - Integrated into the Electronic Chronic Care Management (eCCM) strategy.  *Integration of ePROM into the Kaiku Health App*  - Adapts Kaiku Health App for seamless integration of electronic Patient-Reported Outcome Measures.  - Demonstrates high agreement, ease of use, and satisfaction.  *Patient Reminders for ePROM Completion*  - Sends timely reminders to patients to complete electronic Patient-Reported Outcome Measures.  *Display of Previous Replies and Symptom Summary*  - Facilitates self-care by displaying all previous questionnaire replies.  - Provides a summary of symptom evolution at the end of each questionnaire.  *Symptom Awareness and Guidance*  - Acknowledges increased symptom awareness and provides guidance for empowerment.  *Symptom Selection Screen for PRO-CTCAE™*  - Allows patients to navigate the complete item bank of PRO-CTCAE™.  - Developed collaboratively with patients and a patient representative.  *Integration with Clinical Information Systems*  - Aims for the integration of ePRO data into Clinical Information Systems (CIS).  - Enhances data accessibility, and reduces technological burden.  *Nurse Access to ePRO Data via Email*  - Nurses are prompted to access the application via email for new symptom reports. | Mobile Application  (web-based) |
|  | Gregory M. Gressel. et al ^95^ | 2019 | United States | To establish feasibility and acceptability of PROMIS ePRO integration in a gynecologic oncology outpatient clinic and assess if it can help identify severely symptomatic patients and increase referral to supportive services. | 336 patients in the Gynecologic Oncology Clinic: | Gynecologic cancer | PROMIS® (Patient Reported Outcomes Measurement Information System) ePRO | Identifying and measuring major symptom domains in gynecologic oncology patients | Computer-based |
|  | Jeannine M. Brant, et al ^49^ | 2019 | United States | To determine the perception of patients and providers from patient-reported outcomes | 121 women (51 with gynecologic cancer and 70 with breast cancer) | Breast Cancer, Gynecologic Cancer | Carevive Care Planning System™ | Generating individualized care plans based on electronic patient-reported outcomes (ePROs). | Computer-based |
|  | Kerry N. L. Avery, et al ^81^ | 2019 | United Kingdom | To develop a hospital EHR-integrated ePRO system to improve the detection and management of complications post-discharge following cancer-related surgery | Phase 1: 18 patients, Phase 2: 59 participants who provided 444 complete self-reports | Cancer-related major abdominal surgery | ePRO  self-report questionnaire | - Real-time electronic symptom monitoring  - Integration into hospital EHR  - Clinical algorithms for patient advice and clinician alerts  - Self-management advice - Web-based access | Web-based |
|  | Sasja A. Schepers, et al ^82^ | 2016 | Netherlands | Determine the fidelity of the KLIK method as implemented in outpatient pediatric cancer care | 205 children with newly diagnosed cancer | Pediatric cancer: Leukemias/lymphomas, Solid Tumors, and Brain Tumors | KLIK method | - Monitoring of electronic patient-reported outcomes (ePROs)  - Validation of HRQoL questionnaires  - Generation of electronic PROfiles (ePROfiles) | Web-based |
|  | Joshua R. Niska, et al ^83^ | 2017 | United States | To assess changes in quality of life (QOL) and adverse events (AEs) during radiotherapy (RT) for head-and-neck cancer using electronic patient-reported QOL (PROQOL) data. | 65 patients | Head and neck cancer | Linear Analog Self-Assessment (LASA) | Real-time electronic collection of patient-reported QOL data, visualization of data for clinicians, colour-coding of clinically meaningful changes, and integration of algorithms based on distress management guidelines. | Web-based |
|  | Steven M. Lucas, et al ^84^ | 2017 | United States | To report on the establishment of a unified, electronic PRO infrastructure and | 773 eligible patients, with 688 (89%) enrolled preoperatively | Prostate cancer | MUSIC Patient Reported Outcomes (PRO) | Collects patient-reported outcomes, integrates outcome data among multiple practices, and provides physician and patient-level reports, risk-adjustment techniques, and quality improvement opportunities. | Web-based |
|  | P. Holch, L, et al ^50^ | 2017 | United Kingdom | To develop a system for patients to self-report and manage adverse events (AE) during and after cancer treatment | Patient advocates (N=9), patients (N=13), and staff (N=19) participated in usability testing | Breast, gynaecological, colorectal, pelvic radiotherapy, upper gastrointestinal surgery | eRAPID (electronic patient self-reporting of Adverse-events: Patient Information and aDvice) | - Secure real-time integration with EPR - Patient self-reporting of AE - Immediate automated advice for AE management - Email notifications for severe AE - Data tracking and adherence monitoring | Web-based |
|  | Andreas D. Hartkopf, et al ^51^ | 2017 | Germany | To investigate the willingness, and assess specific needs, and barriers of adjuvant breast cancer (aBC) and metastatic breast cancer (mBC) patients in nonexposed and exposed settings before implementing digital electronic Patient-Reported Outcome (ePRO) | 202 participants (nonexposed group: 96, exposed group: 106) | Breast cancer | ePROCOM (Electronic-Based Patient-Reported Outcome) | -Electronic monitoring of patient-reported outcomes (pro)  -Automated triggers and real-time data capture  -Potential for longitudinal symptom assessment  -Enhanced usability and acceptance  -Tailored educational and support services | Web-based application |
|  | Kate Absolom, et al ^52^ | 2017 | United Kingdom | To improve the safe delivery of cancer treatments, enhance patient care, and standardize adverse event (AE) documentation | Internal pilot phase with 87 participants, full trial target sample of 504 participants | Breast, colorectal and gynaecological cancer | eRAPID (electronic patient self-Reporting of Adverse-events: Patient Information and aDvice) | - Patients can report AEs from home, making it convenient and patient-centered  - Immediate access to AE reports for clinical teams ensures timely interventions  - Generates alerts for severe AEs, prioritizing critical cases for immediate attention  - Provides patient advice for managing mild AEs, promoting self-management and reducing unnecessary hospital visits | Web-based |
|  | Liv Marit Valen Schougaard, et al ^62^ | 2016 | Denmark | To implement telepatient-reported outcomes (telePRO) as the basis for follow-up in chronic and malignant diseases using the generic PRO system AmbuFlex | AmbuFlex was implemented in nine diagnostic groups in Denmark. A total of 13,135 outpatients from 15 clinics have been individually referred.  -Response rates for the initial questionnaire ranged from 81% to 98% in different patient groups | Prostate, colorectal cancer | AmbuFlex | - PRO data collection with mixed-mode (paper or web-based)  -PRO-based automated decision algorithm with defined thresholds  -PRO-based graphical overview for clinical decision support  -Integration of PRO into patient care | Web-based |
|  | Maria K. Peltola, et al ^77^ | 2016 | Finland | Assess the suitability of Kaiku® (an ePRO application) for collecting patient-reported outcomes (PROs) related to early side effects of radiotherapy and health-related quality of life in head and neck cancer (HNC) patients. | Nine HNC patients were approached, and five consented to participate. | Head and neck cancer (HNC) | Kaiku® | - Real-time, online collection of patient-reported outcomes. - Self-assessment of side effects and quality of life. - Communication with the medical team. | Mobile Application  (web-based) |
|  | Beate Mayrbäurl, et al ^63^ | 2016 | Austria | To assess health-related quality of life (HRQOL) in patients with advanced colorectal cancer across different lines of palliative chemotherapy | 100 consecutive patients with colorectal carcinoma | Colorectal cancer | ePRO applications | - The EORTC QLQ-C30 questionnaire to assess multiple aspects of patient quality of life.  - Data was collected electronically using tablet PCs | tablet PCs |
|  | J. Graf, E. et al ^53^ | 2016 | Germany | To determine the extent to which existing computer skills, disease status, health-related quality of life, and sociodemographic factors affect patients' willingness to use electronic methods of data collection (ePRO) | 96 | Breast cancer | ePRO applications | Captures patient quality of life, state of health, computer skills, and willingness to use electronic surveys. | Mobile-based |
|  | Katharina Duregger, et al ^68^ | 2016 | Austria | To develop a concept and implement a prototype for introducing electronic Patient Reported Outcomes (ePRO) into the existing neuroblastoma research network by applying Near Field Communication (NFC) and mobile technology. | N/A | Neuroblastoma (the primary focus of the study), but the system's applicability extends to other pediatric cancers and rare diseases. | - EUPID Mobile (for physicians) - MoKi (telemonitoring system for patients) | - EUPID Mobile for physicians: Enables patient registration, PSN generation, and user credential provision. - MoKi for patients: Facilitates the capture of vital parameters and secure data transmission to OpenClinica. - NFC technology for safe and contactless communication. | Mobile application |
|  | Renee A. Cowan, et al ^85^ | 2016 | United States | The assessment, and feasibility of acceptability and satisfaction of a Web-based system for capturing patient-reported outcomes (PROs) in the immediate postoperative period in gynecologic cancer surgery patients. | 96 eligible patients | Gynecologic cancer | Symptom Tracking and Reporting (STAR) system | - Capture PROs in the immediate postoperative period. - Enable patients to self-report common postoperative symptoms. - Alerts for concerning patient responses. - Enhanced patient empowerment. | Web-based |
|  | L. M. Wintner, et al ^60^ | 2015 | Austria | Assessment, and the feasibility of routine clinic-ePRO/home-ePRO with the Computer-based Health Evaluation System (CHES) software. | - 113 patients for clinic-ePRO - 45 patients for home-ePRO | Gastrointestinal, glioma, gynaecological, lung, neuroendocrine, and testicular cancers | Computer-based Health Evaluation System (CHES) | - Electronic PRO assessment in clinical and home settings - Availability of specialized software (CHES) - Potential for improved symptom management, better identification of psychosocial problems, and enhanced communication between healthcare professionals and patients | Computer-based |
|  | Lynne I. Wagner, et al ^69^ | 2015 | United States | To integrate electronic patient-reported outcome (ePRO) assessment into the electronic health record (EHR) and clinical workflow for symptom screening in ambulatory cancer care. | 636 women receiving gynecologic oncology outpatient care | Ovarian,  Uterine,  Cervical,  Other female genital malignancy | PROMIS Computer Adaptive Tests (CATS) | Real-time symptom reporting, clinician notification of severe symptoms, automated triage for psychosocial, information, and nutritional concerns. | Computer-based |
|  | Erqi L. Pollom, et al ^86^ | 2015 | United States | To evaluate the feasibility of eQOL data collection using a touch-screen tablet device in patients undergoing treatment for head and neck cancer | 50 patients | Head and neck cancers | Qualtrics (secure online survey program) on a touch-screen tablet device (iPadTM) | - Ability to administer electronic Quality of Life surveys using touch-screen tablet devices (iPadTM). - Integration with Qualtrics for data capture, analysis, and de-identification. | Web-based |
|  | Sophia K. Smith, et al ^54^ | 2014 | United States | Demonstrate how an electronic patient-reported outcome (ePRO) system can aid in distress management in oncology. | 17,338 | Breast, lung, and gastrointestinal cancer patients | PACE Software | - Assessment of the "whole person" - Real-time, colour-coded reports - Normalized t scores for distress and despair - Efficient tracking of symptom item changes over time. | Web-based software |
|  | L M Wintner, et al ^61^ | 2013 | Austria | To assess the quality of life (QOL) of lung cancer patients undergoing chemotherapy (CT) across multiple treatment lines. | 187 | Lung cancer | The Electronic Patient-Reported Outcome Monitoring System (Computer-based Health Evaluation System, CHES) was used for QOL assessment. | - The system can collect and store patient-entered data, providing an authorized doctor with a QOL profile for each patient.  - It can be used for longitudinal QOL assessment and tracking changes in QOL during CT. | Computer-based |
|  | August Zabernigg, et al ^74^ | 2012 | Austria | To investigate QOL trajectories from adjuvant treatment to palliative 3rd-line therapy | 80 patients (Pancreatic cancer and cancer of the bile ducts) | Pancreatic cancer and Bile duct Cancer | Electronic patient-reported outcome monitoring (ePROM) | Provides insights into QOL changes during different chemotherapy lines, potential to assess the effects of adjuvant chemotherapy on QOL | Computer-based |
|  | Abernethy, Amy P. MD, et al ^55^ | 2010 | United States | Demonstrate a rapid learning healthcare model in an academic oncology clinic using electronic patient-reported outcomes (ePROs) as foundational data | Metastatic breast cancer (n = 65) and gastrointestinal cancer (n = 113) patients in Duke Cancer Clinics | Breast and gastrointestinal cancer | electronic PROs (ePROs) | Collecting patient-reported symptom data, feasibility of e/Tablet use, the potential for personalized medicine, real-time research-quality data collection | e/Tablet-based ePRO system |
