# Supplementary material for: Electronic patient-reported outcome systems and capabilities in cancer care: a systematic review
Source: Front Digit Health. 2025 Aug 18;7:1560533. doi: 10.3389/fdgth.2025.1560533 (PMC12399665; doi:10.3389/fdgth.2025.1560533)
Supplement: Supplementary file 3 [file Datasheet3.docx]

**Appendix C.** Definition and Usage of Electronic Systems and Platforms

| Number | Electronic Systems and Platforms | Frequency | Definition and Usage |
| --- | --- | --- | --- |
|  | PRO-CTCAE (Patient Reported Outcome of Common Terminology Criteria for Adverse Events) ^18, 19, 30, 37, 40, 66, 72, 73^ | 8 | PRO-CTCAE concentrates on capturing patient-reported outcomes related to the common terminology criteria for adverse events. PRO-CTCAE is a patient-reported outcome (PRO) measure that ascertains in real time the presence, severity and interference of symptoms experienced by patients participating in cancer clinical trials. Real-time ascertainment of symptomatic adverse events using PROs could improve the precision and reproducibility of adverse event reporting. |
|  | CHES (Computer-based Health Evaluation System) ^4, 38, 48, 60^ ^22, 61, 74^ | 7 | The Computer-based Health Evaluation System (CHES) developed by Evaluation Software Development (ESD) provides a convenient software solution for electronic data capture (incl. electronic questionnaire administration and electronic case report forms), storage and data analysis. In addition, our software provides an elaborate system for facilitating the interpretation of patient-reported outcomes (PROs) including graphical real-time feedback. CHES provides a multicomponent approach for PRO assessment and can be easily adapted to the specific requirements of researchers, clinicians, patients, and the local organizational setting. |
|  | eRAPID (Electronic patient self-Reporting of Adverse-events: Patient Information and aDvice) ^7, 41, 50, 52, 64^ | 5 | eRAPID focuses on patients' self-reporting of adverse events. Electronic patient self-Reporting of Adverse-events: Patient Information and aDvice (eRAPID) is web-based and accessible from home or mobile device, for patients to complete symptom reports and receive severity-based advice. Recommendations for self-management are provided for milder symptoms and advice for when to contact the hospital for severe issues. A graphing feature allows patients to review personal symptom data over time. The system includes a facility to notify healthcare teams via email when a severe symptom is reported. Patient-reported data are transferred in real-time to be accessed by health professionals through the hospital's electronic patient records (EPRs) for use in routine consultations and assessments. This system enhances patient engagement by allowing individuals to report their symptoms. Furthermore, eRAPID provides relevant information and advice based on patients' reported experiences, contributing to proactive symptom management and personalized care. |
|  | Noona ^13-15, 35^ | 4 | Noona is a web app and patient outcomes management solution designed to engage patients in their care with real-time symptom reporting and monitoring, streamlined clinical workflows to promote evidence-based care, and access to rich data insights for better management and ongoing assessment throughout care. Noona (Varian Medical Systems, Inc, Palo Alto, CA), a modular digital cancer follow-up application (CFUA), is used to improve communication between patients with cancer and health care providers and to collect data on the patients’ symptoms at different phases of their cancer care. Access to clinical care teams, structured data capture with actionable content, workflow efficiency design, prebuilt treatment modules covering most common cancer types, rapid deployment for immediate patient impact, and real-world evidence and outcomes data for value-based care are some of its features. |
|  | PROMPT-Care (Patient Reported Outcome Measures for Personalized Treatment and Care) ^56, 58, 75^ | 3 | PROMPT-Care is featured in collecting patient-reported outcome measures for personalized cancer treatment and care. PromptCare is a leading provider of respiratory and speciality infusion and pharmaceutical therapies, helping patients with complex, chronic, and rare conditions live their fullest lives at home. Unique Journeys Deserve Personalized Care: For people with chronic and complex health conditions, a personalized plan and network of support are more than just comfort. They’re proven means to a longer life. |
|  | EPIC Electronic Health Record (EHR) ^16, 42^ | 2 | Epic is a cloud-based EHR solution catering to a number of specialities. Epic is in use across a broad range of practices, from community hospitals and independent practices to multi-speciality hospital groups and hospice care providers. Epic provides the standard range of ‘core’ EHR features, and practices can add modules depending on speciality. Epic has a strong focus on patient engagement and facilitating remote care. Epic EHR features include an extensive patient portal, available as a native app for both Android and iOS operating systems, as well as numerous telehealth options - from supporting video visits and post-surgical follow-ups to patient monitoring features. |
|  | AmbuFlex ^62, 76^ | 2 | AmbuFlex serves as a software platform for electronic patient-reported outcome systems. Its application involves efficient data collection and management of patient-reported information. AmbuFlex enhances communication between patients and healthcare providers, contributing to a more comprehensive understanding of patient experiences and needs in cancer care. AmbuFlex is a generic clinical telePRO system for mixed-mode (web and paper) PRO data collection for use in clinical practice. The overall goal is to use PRO for clinical decision support to improve the quality of care, promote patient-centred care, optimise the use of resources in the healthcare system, and use data for research purposes. |
|  | Kaiku® ^66, 77^ | 2 | Personalised digital health interventions for cancer patients: Kaiku Health is a platform for digital health interventions. It provides patient-reported outcome monitoring and intelligent symptom king. The use of Kaiku Health helps cancer patients provide optimised care through timely symptom management and improved workflow. Kaiku creates value for cancer patients, healthcare providers and cancer research: Personalised support and improved quality of life for patients. Kaiku Health is a patient’s companion throughout different phases of therapy. It reduces uncertainty around symptoms by educating patients on their self-management strategies. Digital symptom monitoring can help cancer patients live longer, reduce ER visits and improve their quality of life. Improved symptom management for care teams. Algorithms triage patients’ symptom reports, which helps clinical staff prioritise their daily work. Multidisciplinary care teams have access to a comprehensive decision support dashboard, which enables intervening in early symptom monitoring. Clinics using Kaiku Health report decreased phone call burden and more efficient patient visits and contacts. Real-world data on the effectiveness of therapies for cancer research. Regulatory-grade patient-reported outcomes data combined with clinical data opens significant new opportunities for evaluating the safety profile and effectiveness of treatments and their long-term outcomes in a real-world setting. This enables the development of new, better-targeted ways to treat cancer. Kaiku Health is currently used in clinical routine use by thousands of cancer patients of over 25 different cancer types. |
|  | Electronic Psycho-Oncological and Palliative Screening (ePOS) ^11^ | 1 | ePOS is designed for psycho-oncological and palliative screening. It allows healthcare providers to assess and address psychological and palliative care needs in cancer patients. ePOS (electronic psycho-oncological and palliative screening), consists of 51 items. It includes a total of four questionnaires and two questions regarding the patient’s subjective need for psychological or palliative care support. |
|  | Prostate cancer application ^88^ | 1 | a smartphone-based application for electronic reporting of outcomes by patients with prostate cancer. The developed application can help to accelerate communication with the specialists. It can improve the quality of care, reduce unnecessary treatment visits and side effects, and improve timely data collection. |
|  | ChemoPRO® ^18^ | 1 | ChemoPRO® patient app has been specially designed to help hospitals and other clients maintain contact with their patients in-between visits. Easy to use, it provides an additional layer of reassurance to patients, in case they need to access information quickly or get in touch with a healthcare professional. |
|  | ONCOpatient® ^25^ | 1 | ONCOpatient has been created to help empower cancer patients to manage their symptoms, and medications and also educate them about their illness and treatment. Patients will only be able to access ONCOpatient through an invitation from their care team. The care team will tailor ONCOpatient to the needs of their patient. They will assign ongoing assessments, medications and information that is related to the patient's specific cancer. |
|  | Digital Health Management from Compliance Solutions GmbH ^89^ | 1 | The IT solution Digital Health Management from Compliance Solutions GmbH was used to record and evaluate patient responses. The tablets with SIM cards (mobile internet) were made available to the respective patients for 9 weeks each to document the diaries. The questionnaires had to be started by the clinic staff, who trained the patients in their use. After completion of a 9-week patient diary phase, data had to be exported by the clinic staff and then deleted from the device before the tablet had been given to another patient. Access to other functions of the tablets had been blocked for patients. |
|  | Cureety ^90^ | 1 | Cureety is a groundbreaking solution in the fight against cancer, providing crucial support to patients, caregivers, and researchers. This CE-marked medical device (CE Mark certification to commercialize your medical and in vitro diagnostic devices in the European Union), seamlessly integrated into the digital platform, facilitates remote patient monitoring and has been successfully employed by numerous hospitals in France, benefiting over 2000 cancer patients in the last three years. During the study, patients actively engaged with personalized Adverse Events (AE) questionnaires, aligning with the Common Terminology Criteria for Adverse Events (CTCAE), contributing valuable data. The resulting global health score classified patients into Correct (green), Compromised (yellow), Fragile (orange), or Critical (red) states, prompting tailored interventions. With Cureety, patients receive discreet acknowledgement or therapeutic recommendations based on their condition, ensuring proactive management of Adverse Events and enhancing the overall cancer care experience. |
|  | Electronic self-assessment and care (eSAC) ^70^ | 1 | electronic self-assessment and care (eSAC) is a web-based, patient-reported outcome (PRO) program for people with advanced ovarian cancer. |
|  | The Life App (multifunctional web-based application for rehabilitation management) ^67^ | 1 | The Life App is a multifunctional web-based application designed for rehabilitation management. Life App”, which based on patient’s responses documents changes in wellbeing during 4 weeks of pediatric rehabilitation, and a 12-month follow-up. The “Life App” was designed by ESD (Evaluation Software Development GmbH in Innsbruck, Austria) after years of experience in research, evaluation, teaching and administration both in university and education institutions. The method was described by Holzner et al. in 2012. Life App is based on Computer-Based Health Evaluation Software (CHES). |
|  | PiiA (patient interactively informs doctor) ^32^ | 1 | PiiA is a web-based solution facilitating patients' completion of Health-Related Quality of Life (HRQoL) assessments on a tablet post-anonymized user credentials provision. The electronic Patient-Reported Outcome (ePRO) version of FACT-B, provided by PiiA, demonstrates reliability in assessing HRQoL among patients with metastatic and adjuvant breast cancer. |
|  | InSight Care ^33^ | 1 | InSight Care provides accessible, high-quality mental health services catering to children, adolescents, adults, and their families. It operates under a service model prioritizing trust, respect, confidentiality, and compassion. InSight Care is dedicated to delivering collaborative mental health care in conjunction with consumers' overall health strategies and diverse medical services. |
|  | GIROfit Phase 2 Pilot Trial ^34^ | 1 | GIROfit Phase 2 Pilot Trial is a specialized system designed for a specific clinical trial, likely encompassing patient-reported outcome monitoring, treatment adherence tracking, and research data collection. Its objective is to propel clinical trials forward and enhance comprehension of intervention effects on cancer patients. |
|  | Head and Neck PROs Oncology platform ^91^ | 1 | The Head and Neck PROs Oncology platform, developed as part of the Patient-Reported Information for Strategic Management (PRISM) program at Memorial Sloan Kettering Cancer Center (MSK), is tailored for head and neck cancer care. It serves as a standard of care for all head and neck cancer patients at MSK, facilitating patient-reported outcome assessment and contributing to treatment strategies. |
|  | SAAD (Serial Assessment of Anxiety and Depressive Symptoms in Breast Cancer) ^39^ | 1 | SAAD is a dedicated tool designed for the serial assessment of anxiety and depressive symptoms in breast cancer patients. Implemented as part of the SAAD trial, a prospective, questionnaire-based serial cohort study, this electronic Patient-Reported Outcome (ePRO) platform enables the evaluation of depressive and anxiety symptoms among patients undergoing breast cancer treatment. |
|  | PAINReportIt® ^65^ | 1 | PAINReportIt® is a pioneering software program providing the first computerized extension of the McGill Pain Questionnaire (MPQ). Published in 2003, this interactive software extends the 1970 MPQ, a renowned pain assessment tool, by presenting pain measurement items to respondents through serial display screens accompanied by pop-up prompts. It encompasses a range of pain parameters, including pain location, quality, and intensity, aiding practitioners in meeting regulatory and policy requirements. |
|  | REDCap electronic data capture tools ^80^ | 1 | REDCap stands as a comprehensive system for electronic data capture, encompassing patient-reported outcomes among its functionalities. Widely employed for research endeavours, REDCap facilitates efficient and secure data collection in oncology studies, thereby contributing to the advancement of scientific knowledge and evidence-based practices. |
|  | Strength Through Insight ^92^ | 1 | Strength Through Insight is a medical application developed by Thomas Jefferson University, aimed at gaining insights into the symptom experiences of patients undergoing treatment for prostate cancer. This research-focused app enables participants to complete weekly questionnaires regarding their health and symptoms, with the overarching goal of comprehending variations in symptomatology throughout the treatment process. |
|  | Signant Health's eCOA software solutions on tablet and mobile devices ^46^ | 1 | Signant Health's eCOA software solutions represent a cutting-edge approach to electronic Clinical Outcome Assessments (eCOA) delivered through tablet and mobile devices. This comprehensive technology suite encompasses electronic Patient-Reported Outcomes (ePRO), facilitating complex Clinical-Reported Outcomes (ClinRO), and providing robust tools and reporting features for sponsors and sites. Guided by over 50 clinical and eCOA experts, Signant's SmartSignals eCOA ensures the highest standards in study design, execution, and evidence generation across global clinical trials. |
|  | MyPal ^93^ | 1 | MyPal is a patient-centred palliative care intervention designed for cancer patients, focusing on capturing and recording symptoms accurately. It aims to personalize palliative care delivery by empowering patients and caregivers to communicate effectively with healthcare professionals. |
|  | iPEHOC (Improving Patient Experience and Health Outcomes Collaborative) ^59^ | 1 | iPEHOC aims to develop a common and sustainable measurement system for collecting and reporting patient-reported outcomes in Quebec and Ontario. Its main goal is to reduce symptom burden and improve patient experience of care by incorporating patient-reported outcome results into routine clinical care. |
|  | Healthcare Monitor (HM) ^97^ | 1 | Healthcare Monitor (HM) is an electronic patient-reported outcome measures (ePROMs) structure for the longitudinal follow-up of head and neck cancer (HNC) patients. It supports research and quality improvement efforts while improving overall patient care and transparency in healthcare. |
|  | Medocity Home Health app ^94^ | 1 | Medocity Home Health is a mobile app that enables patients to connect virtually with their care professionals between visits. It provides personalized support for patients' specific needs and health conditions, including secure messaging, medication tracking, symptom reporting, and access to educational resources. |
|  | EirV3 (Eir-Doctor) ^47^ | 1 | EirV3 is an electronic tool for administering patient-reported outcome measures (Eir-Patient) that provides immediate scores to physicians (Eir-Doctor). It emphasizes perceived usability for successful implementation. |
|  | Carevive Care Planning System™ ^49^ | 1 | Carevive Care Planning System™ is designed for comprehensive cancer care planning, including patient-reported outcome monitoring and treatment management. It utilizes a Clinical Intelligence System to personalize care planning and improve patient outcomes. |
|  | KLIK method ^82^ | 1 | The KLIK method is an online system for routine monitoring and discussion of electronic patient-reported outcomes (ePROs) for children with chronic diseases. It enhances discussions of psychosocial functioning during outpatient consultations and improves healthcare provider satisfaction. |
|  | Linear Analog Self-Assessment (LASA) ^83^ | 1 | LASA is a method for linear analogue self-assessment used for efficient assessment of patient-reported experiences in cancer care. It measures various health-related quality-of-life domains using a validated scale. |
|  | MUSIC Patient Reported Outcomes (PRO) ^84^ | 1 | MUSIC PRO is designed for monitoring and improving patient-reported outcomes in radical prostatectomy outcomes. It utilizes a validated questionnaire to assess functional status and quality of life before and after surgery. |
|  | ePROCOM (Patient Reported Outcomes and Compliance Analysis) ^51^ | 1 | ePROCOM is an electronic-based patient-reported outcome system designed for cancer patients. It facilitates outcome monitoring, and data collection, and supports enhanced communication between patients and healthcare providers. |
|  | EUPID Mobile (for physicians) - MoKi (telemonitoring system for patients) ^68^ | 1 | EUPID Mobile for physicians and MoKi for telemonitoring are complementary systems for patient-reported outcome monitoring and remote patient monitoring in cancer care. They aim to optimize communication and care coordination between physicians and patients. |
|  | Symptom Tracking and Reporting (STAR) system ^85^ | 1 | STAR is a web-based interface used for self-reporting symptoms by patients with cancer. It facilitates symptom monitoring using questions adapted from the National Cancer Institute’s Common Terminology Criteria for Adverse Events, supporting efficient and accurate symptom assessment. |
|  | PROMIS Computer Adaptive Tests (CATS) ^69^ | 1 | PROMIS CATs utilize a computer algorithm to administer patient-reported outcome items tailored to symptom severity. It generates reliable and valid symptom scores, contributing to improved assessment of patient-reported outcomes. |
|  | Qualtrics (secure online survey program) ^86^ | 1 | Qualtrics is a secure online survey program used for various purposes, including market research, customer experience, product testing, and employee experience. It provides automated export to common data analysis packages and ensures data security. |
|  | PACE Software ^54^ | 1 | PACE software is an ePRO data collection system comprising software, PRO survey instruments, analytics/reporting, and a process for integration into care. It supports distress screening and outpatient oncology care by efficiently managing and analyzing patient-reported outcome data. |
|  | Vinehealth ^26^ | 1 | Vinehealth is a smartphone application using behavioural science and machine learning for cancer patients' self-management and psychological well-being. It involves patient-reported outcome monitoring, self-management support, and personalized features to enhance cancer care. |
|  | App-Controlled Treatment Monitoring and Support for Patients With Head and Neck ^87^ | 1 | APCOT (App-Controlled Treatment Monitoring and Support for Head and Neck Cancer Patients) trial assesses the feasibility of monitoring head and neck cancer patients during (chemo)radiation therapy using a mobile app. It aims to improve treatment monitoring and support for patients. |
|  | V-Care Platform ^19^ | 1 | V-Care is an eHealth platform comprising an electronic patient-reported outcome (ePRO) system for cancer patients receiving immune checkpoint inhibitors (ICIs). It enables personalized care, better patient management, and improved communication between patients and healthcare providers. |
|  | ePROhub, ePRO-Doctor Client, and ePRO-Patient Client  ^29^ | 1 | ePROhub provides a platform for collecting and managing patient-reported outcome data, while ePRO-Doctor and ePRO-Patient clients serve as interfaces for physicians and patients, respectively. These components support efficient data collection, real-time progress monitoring, and improved patient-provider communication. |
